# Mitigating Water-Deficit Stress in Mediterranean Grapevines: Field Responses of Agiorgitiko and Assyrtiko to a Proline-Rich Yeast Derivative

**DOI:** 10.3390/plants15172631

**Published:** 2026-08-28

**Authors:** Despoina G. Petoumenou, Vasiliki Liava, Athanasia Tsigkreli, Christos Spyridon Dimpertis, Dimitrios Saripavlidis, Emmanouil Kontaxakis, Spyridon Souipas

**Affiliations:** 1 Laboratory of Viticulture, Department of Agriculture, Crop Production and Rural Environment, University of Thessaly, Fytokou Street, 384 46 Volos, Greecesouipas@uth.gr (S.S.); 2 Department of Agriculture, School of Agricultural Sciences, Hellenic Mediterranean University, P.O. Box 1939, 71410 Heraklion, Greece; kontaxakis@hmu.gr

**Keywords:** *Vitis vinifera* L., biostimulants, water stress, photosynthesis, leaf temperature, intrinsic leaf water use efficiency, anthocyanins, total phenolics

## Abstract

Rising temperatures and increasing drought frequency threaten grapevine physiological performance, yield, and berry quality in Mediterranean vineyards. This study evaluated the responses of two field-grown Greek *Vitis vinifera* L. cultivars, the red Agiorgitiko and the white Assyrtiko, to contrasting water regimes and foliar application of a proline-rich yeast derivative over two consecutive growing seasons. Five field treatments were included: a fully irrigated untreated reference (C), deficit-irrigated grapevines with or without yeast application (DI-Y and DI-NY), and rainfed grapevines with or without yeast application (NI-Y and NI-NY). The four water-limited treatments constituted a 2 × 2 factorial subset for formal testing of water condition, yeast application, and their interaction. Vegetative growth and physiological parameters were assessed in 2022, whereas yield and berry composition were evaluated in 2022 and 2023. In Agiorgitiko, significant water × yeast interactions were detected for primary leaf area and shoot length and, during morning measurements, for leaf assimilation rate, transpiration, stomatal conductance, and intrinsic water use efficiency, indicating water condition-dependent responses to the yeast derivative for these traits. In Assyrtiko, such interactions were less evident for vegetative and physiological traits but were pronounced for several berry traits and for total soluble solids. Yield did not show a significant water × yeast interaction in either cultivar, despite numerical increases in NI-Y relative to NI-NY. Leaf temperature responses were mainly associated with yeast and date-dependent effects rather than a consistent water × yeast interaction. Overall, the response to the proline-rich yeast derivative was cultivar- and trait-dependent, supporting its potential as a complementary tool under water limitation while emphasizing the importance of stress level and seasonal conditions.

## 1. Introduction

Projected climate change scenarios indicate an increase in the intensity and frequency of extreme heat events, including heatwaves, together with changes in precipitation patterns that affect both rainfall distribution and intensity [1,2,3]. In viticulture, these changes are expected to increase the incidence of heat, drought, and water deficit episodes, with negative consequences for grapevine phenology, physiological performance, yield, and berry composition [4].

Drought is becoming a major constraint in many agricultural production areas, particularly in the Mediterranean basin, where prolonged dry periods are increasingly common [5]. At the same time, unsustainable water use represents a growing environmental and agronomic concern [6]. Therefore, improving irrigation efficiency and developing water-saving strategies that maintain grapevine productivity and berry quality are key priorities for sustainable viticulture [1,7]. In this context, a better understanding of grapevine water requirements is essential for the development of precise and environmentally sustainable irrigation practices [8,9].

Irrigation requirements vary according to cultivar, soil conditions, climate, and phenological stage. Deficit irrigation is considered an effective strategy to reduce water use, as it can impose controlled water limitation while minimizing adverse effects on vine physiology and yield, and in some cases improving grape composition [7,10,11]. When properly managed, drip irrigation and deficit irrigation strategies may reduce water losses, optimize water uptake, and regulate vine transpiration through stomatal control and canopy growth adjustment [12].

The response of grape composition to water deficit depends on the intensity and duration of stress, as well as on the developmental stage at which it occurs [3]. Regulated deficit irrigation applies water restrictions during specific phenological periods rather than imposing a constant deficit throughout the season, allowing more targeted management of vine water status [10,13]. Periods such as bloom, fruit set to veraison, and veraison to harvest are particularly sensitive to water availability, although their relative importance may vary depending on cultivar and environmental conditions [13]. Previous studies have shown that early-season water deficits, especially from flowering to bunch closure or from fruit set to veraison, may reduce vegetative growth, berry size, and yield more strongly than late-season deficits [14,15].

However, irrigation management alone may not be sufficient under increasingly severe summer stress conditions, and integrated adaptation strategies are gaining importance. Practices such as shade nets, antitranspirants, and delayed pruning have been proposed to improve grapevine resilience to heat and water stress [4]. In parallel, biostimulants have attracted increasing interest as complementary tools for improving plant tolerance to abiotic stress. Foliar applications of kaolin, seaweed extracts, and methyl jasmonate, among other compounds, have been reported to improve grapevine responses under drought and/or heat stress conditions [16,17]. Within this framework, yeast-derived biostimulants have been investigated mainly for their effects on berry and wine composition [18,19,20,21,22,23,24]. Nevertheless, their potential role under contrasting irrigation regimes remains less well documented.

Previous studies have shown that yeast-based formulations may influence grape phenolic composition and quality traits. In Agiorgitiko grapes, the effects of yeast application under different irrigation levels were evaluated in relation to phenolic composition [25], but physiological performance and vine productivity were not assessed. Evidence from other crops also supports the potential of yeast extracts under water deficit. In *Hypericum perforatum* L., foliar yeast application improved biomass, water content, and secondary metabolite accumulation under water-limited conditions [26]. Similarly, in wheat, yeast extract enhanced growth, productivity, and quality under different drought levels, with the strongest responses observed under moderate to severe water deficit [27].

Beyond their effects on fruit and wine composition, yeast-derived compounds have also been reported to influence plant physiological and biochemical responses to abiotic stress in several species. Foliar yeast extract has been associated with reduced oxidative stress markers, such as H_2_O_2_ and malondialdehyde, and with enhanced antioxidant enzyme activity under water deficit, together with improved relative water content, chlorophyll retention, and photosynthetic pigment concentration [26,28,29]. Similar protective effects on growth and yield under drought have also been reported in maize and other field or horticultural crops [30]. These responses suggest that yeast-derived compounds may contribute to osmotic adjustment, antioxidant defense, and maintenance of photosynthetic function under stress conditions, providing a physiological basis for evaluating yeast-derived biostimulants as tools to mitigate water-deficit stress in perennial crops such as grapevine [31].

Despite these findings, information on the combined use of deficit irrigation and yeast-derived biostimulants under open-field vineyard conditions remains limited. Most studies on foliar applications and water deficit have been conducted under controlled or pot conditions, whereas field studies are needed to better evaluate the practical relevance of these approaches [16].

Therefore, the aim of this study was to assess the response of two Greek grapevine cultivars, Agiorgitiko and Assyrtiko, to different water regimes and foliar application of a proline-rich yeast derivative under field conditions. Specifically, the study evaluated vegetative growth, gas exchange, leaf temperature, yield components, and berry composition. The four water-limited treatments (DI-NY, DI-Y, NI-NY, and NI-Y) were analyzed as a 2 × 2 factorial subset to test the effects of water condition, yeast application, and their interaction, while the fully irrigated untreated treatment (C) was retained as a non-stressed reference. The findings may contribute to the development of integrated irrigation and biostimulant management strategies for vineyards exposed to water-limited Mediterranean conditions.

## 2. Results

### 2.1. Meteorological Data

**Figure 1:**
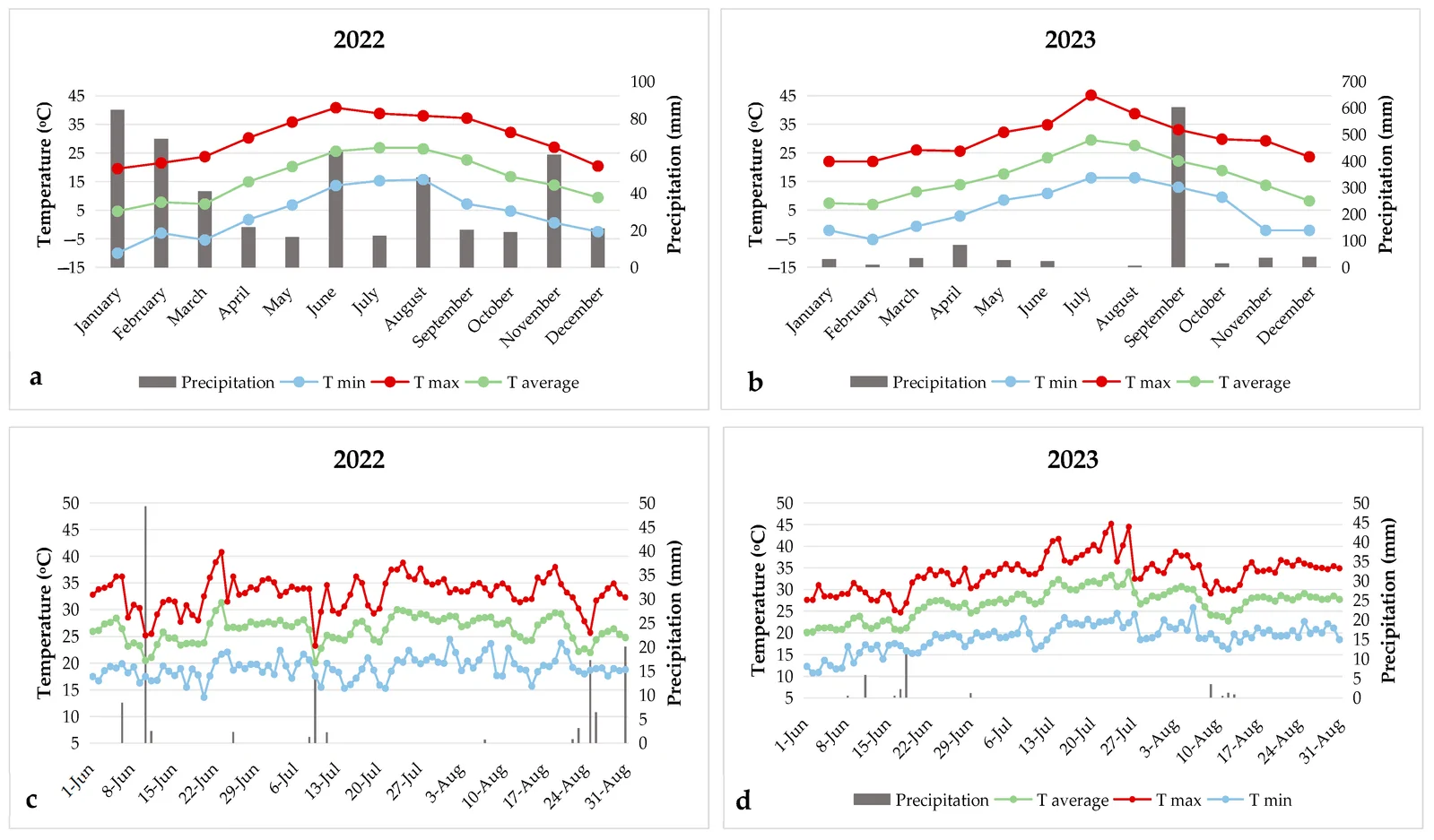
Monthly (**a**,**b**; January–December) and daily (**c**,**d**; June–August) evolution of maximum, mean, and minimum air temperature and precipitation at the experimental location in 2022 (**a**,**c**) and 2023 (**b**,**d**).

Meteorological conditions differed markedly between the 2022 and 2023 growing seasons, resulting in contrasting environmental conditions during grapevine development (Figure 1). In 2022, maximum air temperature reached 41 °C in June, while temperatures remained relatively stable during July and August. Several rainfall events were recorded during the summer period, particularly in June and August.

In contrast, the 2023 growing season was characterized by higher temperatures and lower summer precipitation. Maximum daily air temperature frequently exceeded 35 °C, while during July temperatures surpassed 40 °C on seven days, reaching a maximum of 45 °C. Mean and minimum temperatures were also higher in 2023 than in 2022. Rainfall was limited during the summer of 2023, with no precipitation recorded in July and only 6 mm in August. Therefore, the second growing season was characterized by warmer and drier conditions during the main period of grapevine growth and berry ripening.

An increase in rainfall was recorded in September 2023, further reflecting the high interannual climatic variability observed during the study period.

### 2.2. Leaf Area and Vegetative Growth

Vegetative growth parameters were assessed only in 2022, for logistical reasons detailed in Section 4.4, and differed among treatments in both cultivars (Table 1). In Agiorgitiko, factorial analysis of the four water-limited treatments showed a significant effect of water condition on primary leaf area (*p* = 0.021) and significant yeast effects on primary leaf area (*p* < 0.001), secondary leaf area (*p* = 0.042), and shoot length (*p* < 0.001). Significant water × yeast interactions were detected for primary leaf area (*p* = 0.002) and shoot length (*p* < 0.001), whereas internode diameter was not significantly affected by water condition, yeast application, or their interaction.

The interaction pattern in Agiorgitiko was consistent with the treatment means. NI-NY had the lowest primary leaf area and shoot length, whereas NI-Y increased primary leaf area from 800 to 1623 cm^2^ and shoot length from 54.9 to 102.2 cm. By contrast, the corresponding differences between DI-NY and DI-Y were smaller. Secondary leaf area also increased with yeast application, but its water × yeast interaction was not significant. The fully irrigated untreated treatment was retained as a non-stressed reference and was not included in the factorial interaction test.

In Assyrtiko, primary leaf area was significantly affected by water condition (*p* = 0.003) and yeast application (*p* = 0.011), but the water × yeast interaction was not significant (*p* = 0.115). No significant factorial effects were detected for secondary leaf area, internode diameter, or shoot length. Although NI-Y showed higher primary leaf area and shoot length than NI-NY, the absence of significant interactions indicates that these differences should not be interpreted as evidence of a water condition-dependent yeast effect for these traits.

### 2.3. Gas Exchange and Leaf Temperature

**Figure 2:**
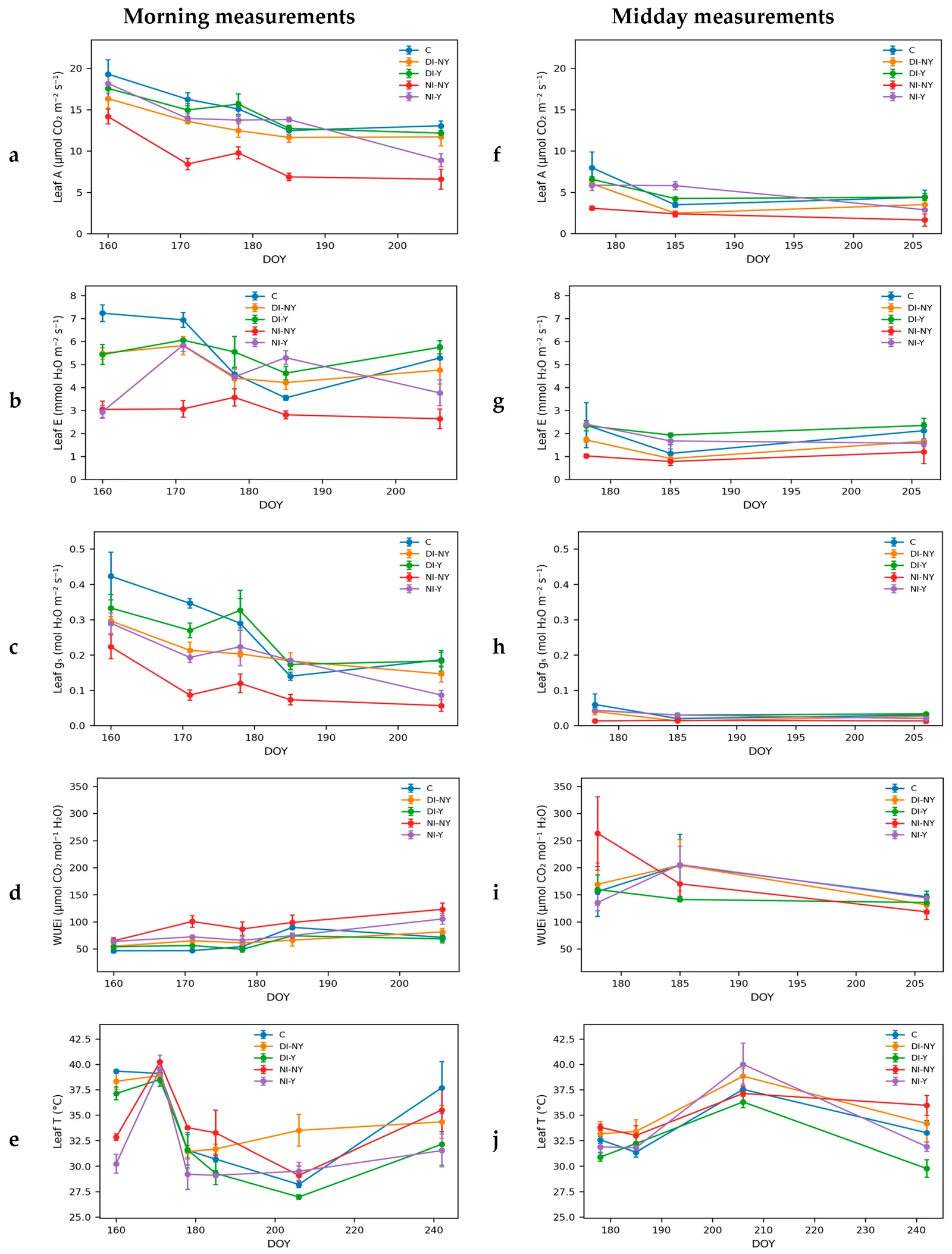
Leaf assimilation rate (A; **a**,**f**), transpiration rate (E; **b**,**g**), stomatal conductance (g_s_; **c**,**h**), intrinsic water use efficiency (WUEi; **d**,**i**), and leaf temperature (Tleaf; **e**,**j**) of field-grown Agiorgitiko grapevines under the five field treatments in 2022. Panels (**a**–**e**) show morning measurements and panels (**f**–**j**) show midday measurements. Points are means ± SE of three treatment plots (*n* = 3). C = fully irrigated untreated reference; DI-NY = deficit-irrigated untreated; DI-Y = deficit-irrigated yeast-treated; NI-NY = rainfed untreated; NI-Y = rainfed yeast-treated. Formal inference for the water-limited factorial subset (DI-NY, DI-Y, NI-NY, NI-Y) was based on repeated-measures analysis including water condition, yeast application, date, and their interactions; detailed *p*-values are reported in Table S1.

Repeated-measures analysis revealed clear water- and yeast-dependent responses in Agiorgitiko during morning gas-exchange measurements (Figure 2; Table S1). Leaf assimilation rate (A) was significantly affected by water condition, yeast application, their interaction, and date (all *p* < 0.001). The significant water × yeast interaction reflected a larger yeast-associated response under rainfed than deficit-irrigated conditions across the measurement period, with NI-NY generally showing the lowest A values after the onset of stronger summer water limitation.

Morning transpiration rate (E) was affected by water condition (*p* < 0.001), yeast application (*p* < 0.001), and their interaction (*p* = 0.012), with additional water × date, yeast × date, and water × yeast × date interactions. Stomatal conductance (g_s_) showed significant water and yeast main effects (both *p* < 0.001) and a significant water × yeast interaction (*p* = 0.015); the three-way interaction with date was also significant (*p* = 0.011). These results indicate that the magnitude of the yeast-associated response varied both with water condition and, for E and g_s_, across measurement dates.

Morning intrinsic water use efficiency (WUEi) was also affected by water condition and yeast application (both *p* < 0.001), with a significant water × yeast interaction (*p* = 0.005), as well as significant date and water × date effects. At midday, however, water × yeast interactions were not significant for A, E, g_s_, or WUEi. Midday A was affected by water condition (*p* = 0.020) and yeast application (*p* < 0.001), E by yeast application (*p* < 0.001), and g_s_ by both water condition (*p* = 0.035) and yeast application (*p* = 0.002), while WUEi showed only a date effect.

Leaf temperature (Tleaf) did not show a significant water × yeast interaction in Agiorgitiko. During morning measurements, Tleaf was affected by yeast application (*p* < 0.001), date, and the water × date interaction; at midday, yeast application (*p* = 0.022) and date were significant. Thus, the lower temperatures observed in some yeast-treated treatments are better interpreted as an overall yeast-associated and temporally variable response rather than as a statistically confirmed stronger effect under one water condition.

Overall, the repeated-measures analysis strengthens the evidence for a water condition-dependent yeast response in Agiorgitiko morning gas exchange, but not for midday gas exchange or leaf temperature. The time-dependent interactions observed for several variables further indicate that treatment effects were not constant throughout the season.

**Figure 3:**
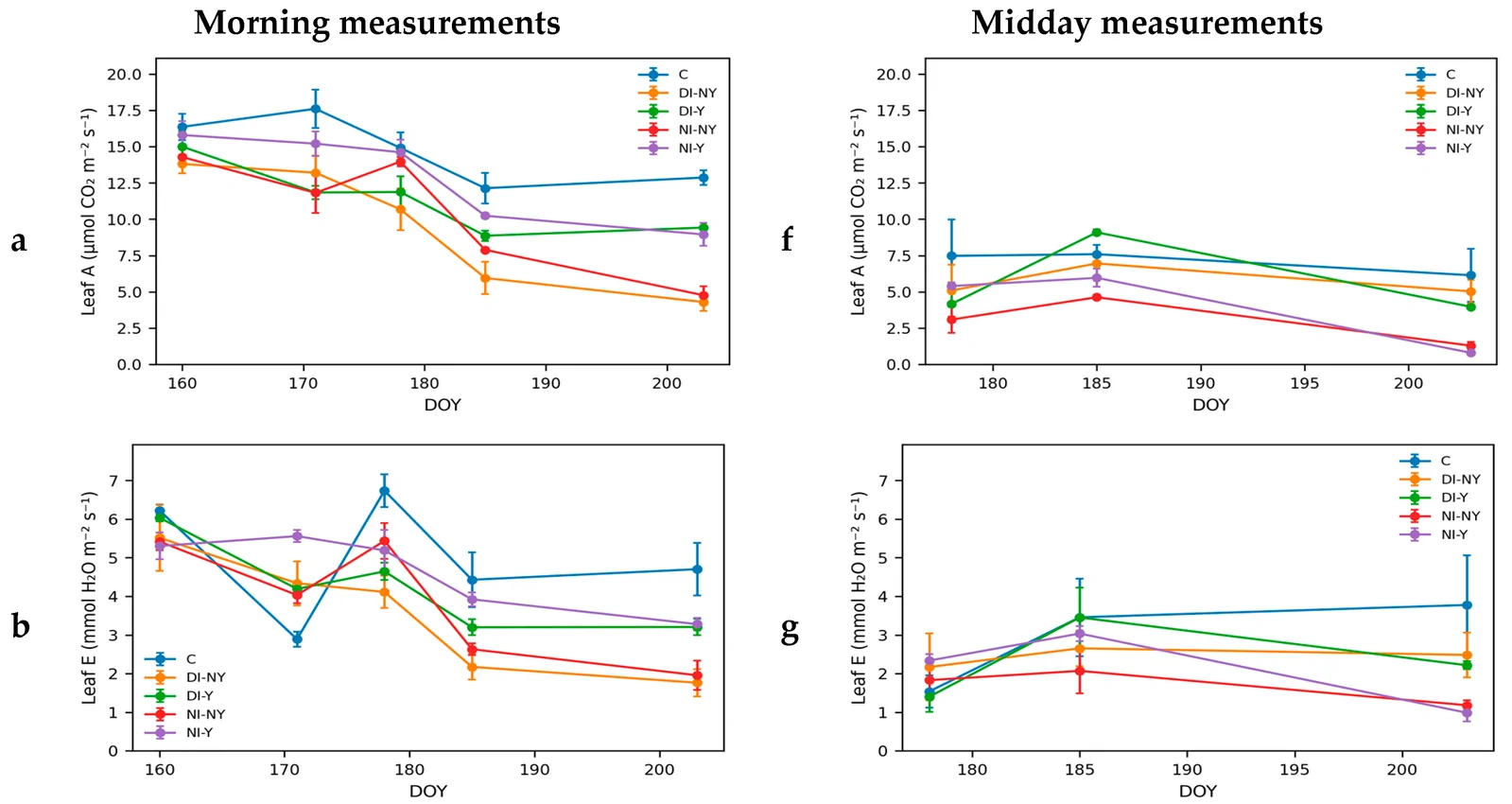

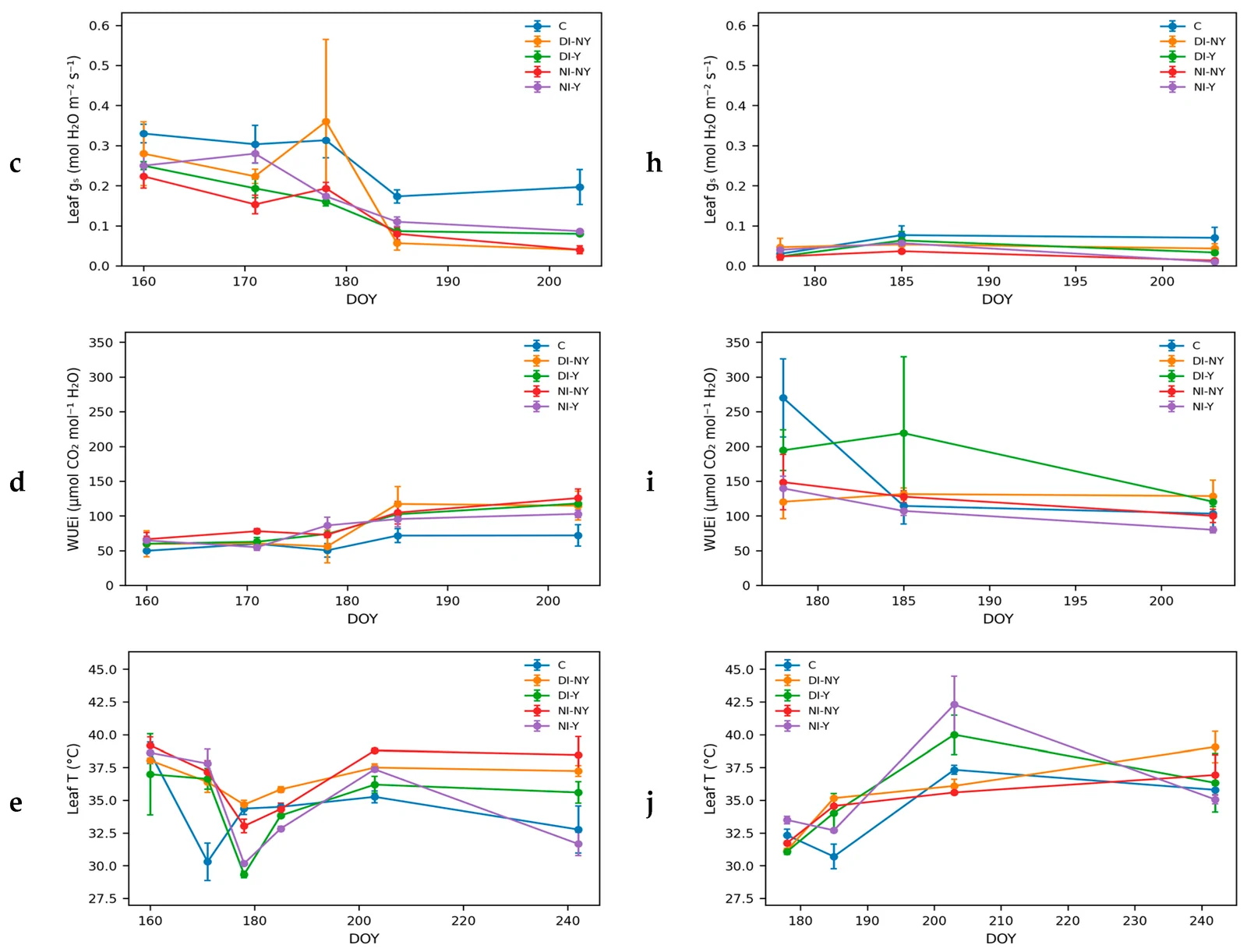
Leaf assimilation rate (A; **a**,**f**), transpiration rate (E; **b**,**g**), stomatal conductance (g_s_; **c**,**h**), intrinsic water use efficiency (WUEi; **d**,**i**), and leaf temperature (Tleaf; **e**,**j**) of field-grown Assyrtiko grapevines under the five field treatments in 2022. Panels (**a**–**e**) show morning measurements and panels (**f**–**j**) show midday measurements. Points are means ± SE of three treatment plots (*n* = 3). C = fully irrigated untreated reference; DI-NY = deficit-irrigated untreated; DI-Y = deficit-irrigated yeast-treated; NI-NY = rainfed untreated; NI-Y = rainfed yeast-treated. Formal inference for the water-limited factorial subset (DI-NY, DI-Y, NI-NY, NI-Y) was based on repeated-measures analysis including water condition, yeast application, date, and their interactions; detailed *p*-values are reported in Table S1.

In Assyrtiko, repeated-measures analysis showed significant morning effects of water condition and yeast application on leaf assimilation rate (A) (water *p* < 0.001; yeast *p* < 0.001) and transpiration rate (E) (water *p* = 0.039; yeast *p* < 0.001), but the water × yeast interaction was not significant for either variable (Figure 3; Table S1). Date significantly affected both variables, and yeast × date interactions were detected for A (*p* = 0.002) and E (*p* = 0.039); E also showed a significant water × yeast × date interaction (*p* = 0.009). These results indicate that yeast-associated physiological responses in Assyrtiko were primarily time-dependent rather than consistently modified by water condition.

Morning stomatal conductance (g_s_) and WUEi did not show significant main effects of water condition or yeast application, nor a significant overall water × yeast interaction. Both varied with date, and g_s_ additionally showed a significant water × yeast × date interaction (*p* = 0.034), indicating that the treatment pattern changed across individual measurement dates without a consistent overall interaction across the season.

At midday, A was significantly affected by water condition (*p* < 0.001), with significant water × date and yeast × date interactions, whereas E showed a water condition effect (*p* = 0.009). Midday g_s_ and WUEi were primarily affected by date. No significant water × yeast interaction was detected for any Assyrtiko midday gas-exchange variable.

Leaf temperature in Assyrtiko likewise showed no significant overall water × yeast interaction. Morning Tleaf was affected by yeast application (*p* < 0.001) and date, with significant water × date, yeast × date, and water × yeast × date interactions. At midday, date and both water × date and yeast × date interactions were significant. Consequently, the temperature response to the yeast derivative was strongly dependent on measurement timing rather than showing a uniform water condition-dependent effect.

### 2.4. Yield Components

In Agiorgitiko, yield was numerically highest in the fully irrigated reference and lowest under rainfed untreated conditions, particularly in 2023 (Table 2). Within the water-limited factorial subset, however, the combined 2022–2023 model showed no significant main effect of water condition or yeast application and no significant water × yeast interaction for yield (*p* = 0.387). A significant Year effect was detected (*p* = 0.027). Thus, the higher numerical yield of NI-Y relative to NI-NY, especially in 2023, should be interpreted descriptively rather than as evidence of a statistically confirmed water condition-dependent yeast effect on yield.

Among cluster traits, cluster length showed a significant water condition effect (*p* = 0.011) and a water × yeast interaction (*p* = 0.027), while cluster width also showed a significant water × yeast interaction (*p* = 0.008). Cluster weight and cluster compactness varied strongly between years (both *p* < 0.001) but did not show significant water × yeast interactions. The number of clusters per grapevine was not significantly affected by water condition, yeast application, or their interaction in the combined model.

Berry weight in Agiorgitiko differed markedly between years (*p* < 0.001) but did not show significant water, yeast, or water × yeast effects in the combined analysis. Berry length, berry width, relative skin mass, and relative seed mass were assessed only in 2022 and were therefore not included in two-year models. In 2022, the only significant factorial interaction among these morphology variables was for relative skin mass (water × yeast, *p* = 0.029).

Selected contrasts with the fully irrigated untreated treatment were used only to assess differences from the non-stressed reference and are reported separately in Table S2. Nonsignificant contrasts were not interpreted as evidence of equivalence or non-inferiority.

In Assyrtiko, yield also showed no significant main effect of water condition or yeast application and no significant water × yeast interaction in the combined two-year model (Table 3). The Year effect approached but did not reach the 0.05 threshold (*p* = 0.057). Numerically, NI-Y yielded more than NI-NY in both seasons, but this difference does not constitute evidence of a differential yeast effect between deficit-irrigated and rainfed conditions.

Year significantly affected the number of clusters per grapevine (*p* = 0.030), cluster weight (*p* < 0.001), cluster width (*p* < 0.001), and cluster compactness (*p* < 0.001). Cluster compactness also showed a yeast × year interaction (*p* = 0.009). In contrast, the combined model did not detect a significant water × yeast interaction for yield, cluster number, cluster weight, cluster length, cluster width, or cluster compactness.

Berry morphology showed stronger factorial responses. Berry weight was significantly affected by water condition (*p* < 0.001) and yeast application (*p* = 0.001), with strong Year and yeast × Year effects and a significant water × yeast × Year interaction (*p* = 0.003), although the overall water × yeast interaction was not significant (*p* = 0.077). Berry length and berry width showed significant water × yeast interactions (*p* = 0.004 and *p* < 0.001, respectively), together with several Year-related interactions.

Relative skin mass was primarily affected by Year (*p* < 0.001). Relative seed mass showed a significant water condition effect (*p* = 0.034) and a strong water × yeast interaction (*p* < 0.001). Significant Year, water × Year, and yeast × Year effects were also detected, whereas the water × yeast × Year interaction was not significant (*p* = 0.157), indicating that the overall interaction pattern was not statistically different between seasons.

### 2.5. Quality Traits

In Agiorgitiko, berry composition showed significant yeast and year effects, but water × yeast interactions were generally absent (Table 4). TSS was affected by yeast application (*p* = 0.015) and Year (*p* = 0.001), with no significant water × yeast interaction. Across the treatment means, yeast-treated water-limited treatments generally had lower TSS than their untreated counterparts, particularly in 2022, but the magnitude of this response did not differ significantly between deficit-irrigated and rainfed conditions.

Titratable acidity (TA) showed a strong yeast effect (*p* < 0.001) and Year effect (*p* < 0.001), together with a yeast × Year interaction (*p* = 0.010). Must pH was mainly season-dependent, with significant Year (*p* < 0.001), yeast × Year (*p* = 0.011), and water × yeast × Year (*p* = 0.008) effects. Thus, the influence of yeast application on acidity-related traits differed between seasons rather than showing a consistent water × yeast interaction.

Total phenolic content was affected by yeast application (*p* = 0.033), while no significant water × yeast interaction or Year effect was detected. Total anthocyanin content showed no significant factorial effect in the combined analysis; the water condition effect approached significance (*p* = 0.055) but did not meet the predefined threshold. Consequently, the treatment differences visible within individual years should be interpreted in the context of the combined two-year model.

Overall, Agiorgitiko berry composition was influenced more consistently by yeast application and interannual variability than by a water condition-dependent yeast response. The strongest evidence for differential yeast effects between the two water-limited regimes in this cultivar was therefore observed in vegetative growth and morning physiology rather than in berry composition.

In Assyrtiko, TSS showed one of the clearest water condition-dependent responses to yeast application (Table 5). Water condition, yeast application, water × yeast interaction, and Year were all significant (*p* < 0.001), whereas interactions with Year were not. In both seasons, the yeast-associated reduction in TSS was pronounced under rainfed conditions (NI-Y vs. NI-NY) but small under deficit irrigation, producing a consistent water × yeast interaction across years.

Titratable acidity did not show significant overall Water, Yeast, or water × yeast effects, but it was strongly affected by Year (*p* < 0.001), with significant yeast × Year and water × yeast × Year interactions (both *p* < 0.001). This indicates that the effect of yeast application on TA changed between seasons and depended on water condition in a year-specific manner.

Must pH did not show significant Water, Yeast, or water × yeast main effects, but the yeast × Year interaction was significant (*p* = 0.015). Total phenolic content showed a yeast main effect (*p* = 0.028) and strong Year-related interactions, including water × Year, yeast × Year, and water × yeast × Year (all *p* < 0.001). These results confirm that the phenolic response to the yeast derivative in Assyrtiko was strongly season-dependent.

The fully irrigated untreated treatment was used as a non-stressed reference rather than as part of the factorial interaction analysis. Accordingly, similarities in mean values between individual water-limited treatments and C are reported descriptively or through planned contrasts (Table S2) and are not interpreted as evidence of statistical equivalence.

Overall, Assyrtiko exhibited pronounced water × yeast interactions for TSS and several berry morphology traits, whereas its gas-exchange responses showed little evidence of a consistent water × yeast interaction across the season. This contrast emphasizes the trait-specific nature of the response to the yeast derivative.

## 3. Discussion

### 3.1. Vegetative Growth Responses to Water Limitation and Yeast Application

Previous studies have shown that irrigation level can strongly influence grapevine vegetative growth. Torres et al. [1] reported that full irrigation enhanced vegetative development in Cabernet Sauvignon, particularly leaf area index and total leaf biomass. Similarly, Pérez-Álvarez et al. [32] found that full irrigation increased leaf area per grapevine, especially in warmer and drier years, and this may partially explain the greater shoot length. In contrast, Cabral et al. [2] reported limited effects on total leaf area in Touriga Nacional across three seasons, except for an increase in shoot number under the highest irrigation level during the third year. More broadly, Uriarte et al. [33], in a meta-analysis, confirmed that increasing water deficit reduces vegetative growth, as reflected by decreases in leaf area and pruning weight.

In the present study, water limitation reduced vegetative growth, particularly under rainfed conditions, but the factorial analysis showed that the yeast response was cultivar- and trait-dependent. In Agiorgitiko, significant water × yeast interactions for primary leaf area and shoot length formally demonstrate that the effect of yeast application differed between deficit-irrigated and rainfed conditions for these traits. The treatment means indicate that the yeast-associated increase was substantially larger under rainfed conditions. In contrast, no significant water × yeast interaction was detected for the vegetative traits of Assyrtiko, although primary leaf area showed significant water and yeast main effects.

Biostimulant applications have been reported to alleviate growth reductions caused by water deficit in grapevines and other crops. For example, root application of a protein hydrolysate biostimulant restored shoot growth in water-stressed Sauvignon Blanc grapevines by promoting the development of shoot tips, with stronger effects in younger leaves [34]. In olive trees, biostimulants did not significantly affect leaf dry weight or leaf area under optimal irrigation, but under water stress, glycine betaine, microalgae, and seaweed extract increased leaf area by 56%, 26%, and 44%, respectively [35]. These findings support the view that different classes of biostimulants may partly compensate for stress-induced reductions in vegetative growth. The present factorial results extend this interpretation by showing that, at least for primary leaf area and shoot length in Agiorgitiko, the response to the proline-rich yeast derivative depended significantly on the level of water limitation.

### 3.2. Gas Exchange, Leaf Temperature, and Water Use Efficiency

Gas exchange responses differed between Agiorgitiko and Assyrtiko. Overall, rainfed conditions tended to reduce photosynthetic performance, transpiration, and stomatal conductance, in agreement with previous studies showing that non-irrigated or deficit-irrigated grapevines generally exhibit lower net photosynthesis and stomatal conductance than fully irrigated grapevines [10,36]. Kucukbasmaci and Sabir [7] also reported reduced stomatal conductance under deficit irrigation, highlighting the sensitivity of stomatal regulation to water availability. Similarly, Zufferey et al. [36] observed a sharp decline in photosynthetic activity and stomatal conductance in non-irrigated grapevines toward the end of the season, indicating the cumulative effect of prolonged water limitation. These responses are commonly associated with stomatal closure, which limits water loss but may also restrict carbon assimilation [10].

In Agiorgitiko, the repeated-measures analysis provided formal evidence that the physiological response to the yeast derivative depended on water condition during morning measurements. Significant water × yeast interactions were observed for A, E, g_s_, and WUEi. The mean patterns were consistent with a larger yeast-associated response under rainfed conditions for several measurements, whereas differences between DI-Y and DI-NY were generally smaller. Importantly, these interactions were not reproduced at midday, demonstrating that the water condition-dependent response was also dependent on measurement period.

In Assyrtiko, the statistical pattern was different. Morning A and E showed significant water and yeast main effects, but no overall water × yeast interaction, while g_s_ and WUEi also lacked significant water × yeast interactions. Several yeast × date and higher-order date interactions were detected, indicating that the response varied during the season rather than showing a stable difference between deficit-irrigated and rainfed conditions. This is consistent with the later-season increases in photosynthetic activity observed in some yeast-treated treatments, but it does not support a generalized stronger yeast effect under rainfed conditions for Assyrtiko gas exchange.

Midday measurements further emphasized this distinction. In Agiorgitiko, midday A, E, and g_s_ showed significant water and/or yeast main effects, but no significant water × yeast interaction. In Assyrtiko, midday A and E were primarily affected by water condition, while g_s_ and WUEi were mainly date-dependent. Thus, the repeated analysis indicates that the clearest factorial interaction between water condition and yeast application occurred in Agiorgitiko morning physiology rather than uniformly across cultivars or times of day.

The enhancement of stomatal conductance in Agiorgitiko, and to a lesser extent in Assyrtiko, under rainfed conditions suggests that yeast derivative application may help maintain gas exchange during water scarcity. The effect of proline-rich yeast derivatives on grapevine gas exchange remains relatively limited in the literature, although recent evidence supports potential benefits under water-limited conditions. In previous studies, the application of biostimulants and other compounds alleviated the negative effects of deficit irrigation. For instance, auxin application significantly increased photosynthesis, stomatal conductance, and intercellular CO_2_ concentration in Rashe and Fakhri in a pot experiment [33]. Similarly, the application of *Ascophyllum nodosum* extract under water deficit conditions increased photosynthesis and stomatal conductance of Pinot noir in a pot experiment [34]. Additionally, outside grapevine, foliar spraying of yeast extract has been shown to enhance photosynthetic efficiency and water content in medicinal plants (*Hypericum perforatum*) [26]. These findings support the potential of yeast derivatives to mitigate stress-induced declines in photosynthetic performance.

Leaf temperature provided complementary information on grapevine physiological status. In both cultivars, Tleaf changed strongly with measurement date, and yeast application affected morning Tleaf in both cultivars and midday Tleaf in Agiorgitiko. However, no significant overall water × yeast interaction was detected for Tleaf in either cultivar or time period. The lower leaf temperatures observed in some yeast-treated water-limited treatments may therefore reflect a yeast-associated and temporally variable effect rather than a statistically confirmed preferential response under rainfed conditions. Del Zozzo et al. [24] similarly reported that a proline-rich yeast derivative decreased maximum leaf temperature during later phenological stages.

Intrinsic water use efficiency (WUEi) also differed between cultivars. In Agiorgitiko morning measurements, WUEi showed significant water, yeast, and water × yeast effects, together with a water × date interaction, indicating that the balance between carbon assimilation and stomatal conductance responded differently to yeast application under the two water-limited regimes. In Assyrtiko, WUEi was mainly date-dependent and showed no significant water × yeast interaction. These contrasting responses further support cultivar-specific physiological strategies under water limitation.

### 3.3. Yield Components and Productivity

Yield responses were affected by water availability and varied between years and cultivars. The contrasting weather conditions between 2022 and 2023 likely contributed to the observed differences in yield performance. In 2023, prolonged heatwaves and limited summer precipitation during key phenological stages probably intensified water limitation, especially in the rainfed treatments. Previous studies have similarly reported that seasonal rainfall and year-to-year climatic variability can strongly influence grapevine yield responses to irrigation [2,10].

Rainfed conditions reduced yield and cluster weight, in agreement with studies showing that deficit irrigation or non-irrigated conditions often decrease yield compared with full irrigation, mainly through reductions in cluster and berry weight [1,6,10,15,32,37]. Calderan et al. [9] reported that water deficit caused a 20–28% reduction in berry mass, accompanied by decreases in skin and seed mass. However, berry tissue responses may vary depending on cultivar and stress intensity; for example, Triolo et al. [38] observed that berries under water deficit were smaller and had a higher skin-to-flesh ratio.

The severity of yield losses depends on both the intensity and timing of water stress. Uriarte et al. [33] reported that mild deficit may even increase yield, whereas moderate or severe deficit generally reduces yield and berry weight. In addition, Caruso et al. [15] and Palai et al. [39] showed that pre-veraison water deficit can have stronger negative effects on berry weight and yield than post-veraison deficit. In the present study, yield reductions were more pronounced under rainfed conditions than under deficit irrigation, supporting the importance of maintaining a minimum level of water supply during critical stages of grapevine development.

In both cultivars, NI-Y showed numerically higher yield than NI-NY, and the relative difference was particularly visible in the drier 2023 season. However, the formal combined analysis showed no significant water × yeast interaction for yield in either Agiorgitiko or Assyrtiko. The yield response to the yeast derivative should therefore be described as a numerical treatment pattern rather than as statistical evidence that yeast more effectively mitigated yield loss under rainfed than deficit-irrigated conditions. Significant interactions were instead detected for selected cluster and berry morphology traits, indicating that treatment effects were expressed more clearly in some yield components than in total yield itself.

The positive effects of biostimulants on yield have been associated with the presence of bioactive compounds that may influence physiological activity, nutrient uptake, and assimilate allocation [40,41]. Del Zozzo et al. [24] reported that a proline-rich yeast derivative increased yield, cluster weight, and berry weight in a non-irrigated Barbera vineyard. However, studies with proline alone did not report consistent effects on berry weight in Tempranillo [42,43] or on bunch and berry traits in Red Globe [44]. Petoumenou and Patris [22] also found that inactivated wine yeast increased yield and berry size in irrigated Crimson Seedless grapevines. Taken together, these findings suggest that the responses observed in the present study are more likely related to the yeast derivative formulation as a whole rather than to proline alone and that their expression may depend on cultivar, water availability, and season.

### 3.4. Berry Composition and Phenolic Responses

Berry composition was influenced by water regime, yeast application, cultivar, and year. In many studies, moderate to severe water deficit has been associated with increased sugar concentration, often due to advanced ripening, reduced berry size, or concentration effects [6,10,11,15,32,38]. Pérez-Álvarez et al. [32] reported that decreases in berry weight and yield under rainfed and deficit irrigation conditions were associated with more advanced maturity. However, the response of TSS to water deficit is not always consistent. Uriarte et al. [33] reported that severe water stress may reduce TSS due to limitations in photosynthesis, while other studies found minimal or inconsistent effects of irrigation on TSS [2,36]. These differences highlight the importance of cultivar, berry size, harvest timing, and seasonal conditions.

In the present study, TSS responses differed markedly between cultivars. In Agiorgitiko, yeast application had a significant overall effect on TSS, but the water × yeast interaction was not significant, indicating that the reduction in TSS associated with yeast treatment was not statistically different between deficit-irrigated and rainfed conditions. In Assyrtiko, by contrast, Water, Yeast, and water × yeast effects were all highly significant, and the interaction was consistent across the two seasons. The treatment means show that yeast application strongly reduced TSS under rainfed conditions, whereas the difference between DI-Y and DI-NY was small. This provides formal evidence that the effect of the yeast derivative on sugar accumulation in Assyrtiko depended on water condition. Similar reductions in TSS after yeast derivative application were reported by Del Zozzo et al. [24] in Barbera under rainfed conditions.

Titratable acidity and pH were strongly influenced by year and by year-dependent yeast responses. In Agiorgitiko, TA showed significant yeast and Year effects together with a yeast × Year interaction, whereas pH showed significant Year, yeast × Year, and water × yeast × Year effects. In Assyrtiko, TA was dominated by Year and significant yeast × Year and water × yeast × Year interactions, while pH showed a yeast × Year interaction. These results indicate that acidity-related responses to the yeast derivative were not uniform but depended substantially on seasonal conditions.

Phenolic and anthocyanin responses were also cultivar-dependent. In Agiorgitiko, total phenolic content showed a significant yeast effect without a significant water × yeast interaction, whereas total anthocyanin content did not show a significant factorial effect in the combined analysis. Although higher anthocyanin concentrations were observed in some rainfed treatments within individual years, these differences should not be generalized beyond the combined model. Kogkou et al. [25] reported that yeast derivative application increased phenolic compounds in Agiorgitiko grapes under deficit irrigation, supporting the potential of yeast-derived formulations to modulate phenolic metabolism, but the present results indicate that this response was not consistently water condition-dependent.

In Assyrtiko, total phenolic content showed a significant yeast effect together with strong Year-related interactions, including water × Year, yeast × Year, and water × yeast × Year. This confirms that the phenolic response changed markedly between seasons and depended on the seasonal combination of water condition and yeast application. Previous studies have likewise reported variable effects of inactive yeast or yeast-derived applications on grape composition, with some studies showing little effect on TSS, TA, or pH [18,20,21,23], while others reported cultivar- and year-dependent changes [19,24,44].

The response to foliar proline-rich formulations may also depend on vine nitrogen status, since proline itself represents a nitrogen-containing compound and its foliar effects can interact with endogenous nitrogen metabolism [43,44,45]. Recent pot-based evidence in Chardonnay under different irrigation regimes also showed that a proline-rich specific yeast derivative improved grapevine water status and enabled reduced irrigation volumes [45]. Vine nitrogen status was not assessed in the present trial, and this represents a limitation when comparing yeast-derivative responses across cultivars, seasons, or vineyards with contrasting soil nitrogen availability. Furthermore, as the LalVigne ProHydro™ formulation contains polysaccharides, peptides, amino acids, and vitamins in addition to proline (Section 4.3), the present study cannot separate proline-specific effects from the effects of the complete yeast-derived formulation. This distinction would require a design comparing pure proline against the complete yeast derivative, which was outside the scope of the present study.

### 3.5. Practical Implications and Future Perspectives

In this study, soil moisture measurements were used to verify the intended differentiation among irrigation regimes rather than to characterise vine water status directly, because the relationship between soil water content and plant water status is not strictly linear and can be modulated by cultivar-specific rooting depth, hydraulic behaviour, and evaporative demand. Physiological measurements of gas exchange and leaf temperature provide a closer approximation of plant water status in the present study; nonetheless, future work should incorporate direct water status indicators, such as stem water potential, to strengthen this link. The physiological interpretations based on gas exchange, leaf temperature, and vegetative growth are based on a single growing season and should be treated as indicative rather than confirmed across contrasting climatic conditions.

Exogenous proline and proline-related compounds may contribute to cellular osmotic adjustment and membrane or protein stabilization under water deficit, potentially helping maintain turgor and stomatal function. This is consistent with some of the treatment-wise patterns observed in the present study, including higher stomatal conductance and lower leaf temperature in yeast-treated grapevines under specific water-limited conditions. Proline and yeast-derived peptides or polysaccharides have also been associated with mitigation of oxidative stress under drought in other crops, which may partly explain the maintenance of photosynthetic activity observed in some yeast-treated rainfed grapevines. However, these mechanisms were not directly measured in the present study and should therefore be considered as plausible explanations rather than confirmed mechanisms.

As reported in Section 4.3, the yeast derivative was applied five times in 2022 (DOY 158, 170, 187, 192, and 201/203, the latter two extra applications triggered by rainfall shortly after the first treatment) but only three times in 2023 (DOY 158, 171, 185). This difference in application number is a potential confound when comparing yeast efficacy between the two seasons. The higher application frequency in 2022, partly related to rainfall events that may have reduced product persistence on the leaf surface, may have contributed to differences in yeast-associated responses between years, independently of seasonal differences in water-stress severity. Moreover, the formulation used in our study contains polysaccharides, peptides, amino acids, and vitamins beyond proline; therefore, the observed effects should be attributed to the whole formulation, and mechanistic attribution to proline specifically cannot be isolated from the present design. Seaweed extracts, such as *Ascophyllum nodosum*, act largely through hormone-like compounds, including cytokinin-, auxin-, and betaine-like activity, that influence stomatal regulation and antioxidant metabolism, as reported by Salvi et al. [40] in Pinot noir under deficit conditions. This differs from the more osmoprotective and nitrogen compound-based mechanism proposed for proline-rich yeast derivatives. Protein hydrolysates primarily supply amino acids and peptides that support nitrogen metabolism and can stimulate root and shoot growth directly, as shown by Meggio et al. [34] in Sauvignon Blanc, which is more growth-promoting or nutritional in nature compared with the mechanism proposed for proline-rich yeast derivatives.

Del Zozzo et al. [24], who tested the same commercial product on field-grown rainfed Barbera, reported increased yield, cluster weight, and berry weight, reduced TSS, improved net photosynthesis and stomatal conductance, and reduced maximum leaf temperature following yeast application. These findings are broadly consistent with several responses observed in the present study. The factorial reanalysis, however, shows that the response was not uniform across traits or cultivars. Significant water × yeast interactions were evident for key vegetative and morning physiological traits in Agiorgitiko and for TSS and several berry morphology traits in Assyrtiko, whereas yield and leaf temperature did not show significant overall water × yeast interactions.

Overall, the results suggest that foliar application of the proline-rich yeast derivative may partly support grapevine performance under water-limited conditions, but the magnitude and statistical expression of the response depended on cultivar, trait, measurement period, and season. The evidence therefore supports a context-dependent effect rather than a uniform benefit across all water-limited conditions. In particular, the strongest formal evidence for water condition-dependent yeast responses was found in Agiorgitiko vegetative growth and morning gas exchange and in Assyrtiko TSS and selected berry morphology traits.

From a practical perspective, the product may be considered as a complementary tool for vineyards exposed to water limitation rather than as a replacement for irrigation management. The experimental design allowed a formal 2 × 2 factorial comparison within the water-limited subset (deficit irrigation vs. rainfed × untreated vs. yeast-treated). However, because a fully irrigated + yeast treatment was not included, the study cannot determine the effect of the product under non-stressed conditions or test a complete factorial structure across the full irrigation gradient.

The results also indicate that the yeast derivative response was expressed through different trait groups in the two cultivars. Physiological measurements in 2022 demonstrated clear water × yeast interactions for Agiorgitiko morning gas exchange, whereas the two-year yield data did not show a corresponding interaction for total yield. In Assyrtiko, the clearest interaction was observed for TSS and selected berry morphology traits. This separation between physiological, productivity, and composition responses is agronomically relevant and argues against using a single outcome variable to characterize biostimulant effectiveness.

Future field studies should include a wider range of cultivars, sites, and stress intensities and, where feasible, a fully irrigated + yeast treatment to extend the present 2 × 2 water-limited factorial analysis to the complete irrigation gradient and to quantify responses under non-stressed conditions. Additional measurements of grapevine water status, soil water dynamics, chlorophyll fluorescence, osmotic adjustment, antioxidant activity, or stress-related metabolites would also help clarify the physiological mechanisms involved and define the conditions under which such applications are most effective.

## 4. Materials and Methods

### 4.1. Plant Material

The experiment was conducted during two consecutive growing seasons, 2022 and 2023, at the experimental vineyard of the University of Thessaly in Velestino, Thessaly, Greece (39°23′42.6″ N, 22°45′27.2″ E). Seven-year-old grapevines of the Greek cultivars Agiorgitiko and Assyrtiko (*Vitis vinifera* L.), both grafted onto 1103 Paulsen rootstock, were used.

Agiorgitiko is the most widely planted red grape cultivar in Greece, whereas Assyrtiko, originally from Santorini, is one of the most internationally recognized Greek white grape cultivars. Grapevines were spaced 1.0 m within the row and 3.5 m between rows and were trained to a bilateral Royat cordon, with 14–16 buds retained per grapevine after winter pruning. The soil was classified as silt loam, with a pH of 7.7 and an organic matter content of 1.95%.

### 4.2. Experimental Design and Treatments

The experiment was established across three spatial blocks per cultivar, each containing all five treatments. Within each block, each treatment plot consisted of five adjacent grapevines, resulting in a total of 15 vines per treatment per cultivar. Treatments were randomly allocated to plots within each block using a random number sequence.

This layout was designed to evaluate whether foliar application of a proline-rich yeast derivative could mitigate the effects of water limitation under field conditions. Yeast application was therefore included under deficit-irrigated and rainfed conditions, while fully irrigated untreated grapevines served as a non-stressed reference. The absence of a fully irrigated + yeast treatment prevents a complete factorial analysis across all three water levels and does not allow the yeast effect to be evaluated under non-stressed conditions. However, the four water-limited treatments (DI-NY, DI-Y, NI-NY, and NI-Y) form a valid 2 × 2 factorial subset, allowing for formal testing of water condition (deficit irrigation vs. rainfed), yeast application (untreated vs. yeast-treated), and their interaction within water-limited conditions.

Five field treatments were compared: 1. fully irrigated untreated vines, maintained at approximately 90% of available soil water (C); 2. deficit-irrigated vines, maintained at approximately 40% of available soil water, without yeast application (DI-NY); 3. deficit-irrigated vines, maintained at approximately 40% of available soil water, with yeast application (DI-Y); 4. rainfed vines without irrigation and without yeast application (NI-NY); 5. rainfed vines without irrigation and with yeast application (NI-Y).

Soil water content was monitored periodically using a WaterScout SMEC 300 soil moisture sensor (Spectrum Technologies, Inc., Aurora, IL, USA). Sensors were installed at a depth of 40 cm within the vine row and measurements of volumetric soil water content (VWC, %) were obtained separately for each irrigation treatment in each of the three experimental blocks. Soil moisture measurements were used to monitor differences in soil volumetric water content among irrigation regimes throughout the growing season. Meteorological data, including mean, minimum, and maximum air temperature and precipitation, were recorded daily by a weather station located within the experimental vineyard.

### 4.3. Yeast Derivative Application and Irrigation Management

The commercial biostimulant LalVigne ProHydro™ (Lallemand Inc., Montreal, QC, Canada) was applied as a foliar treatment (at the manufacturer’s recommended dose of 1 kg ha^−1^) only to the DI-Y and NI-Y treatments. Three applications were performed at the grapevine phenological stages J (berry set), K (berry growth), and L (veraison), according to the extended BBCH scale [46]. The spray solution was applied using a hand-held backpack sprayer to ensure uniform canopy coverage. No adjuvant was added to the spray solution. All applications were carried out early in the morning under comparable weather conditions, avoiding periods of strong wind, rainfall, and high evaporative demand to maximize spray deposition and foliar uptake. The LalVigne ProHydro™ product is a specific yeast derivative based on *Saccharomyces cerevisiae* and contains L-proline derived from *Corynebacterium glutamicum*. Although the formulation is marketed for its proline content, yeast derivatives may also contain other bioactive compounds, including polysaccharides, peptides, amino acids, vitamins, and other metabolites, which may contribute to plant responses under abiotic stress. Therefore, the observed effects were interpreted as responses to the whole formulation rather than to proline alone as an isolated compound.

In 2022, the first application was carried out on day of year (DOY) 158 (7 June; berry set). A second application was performed on DOY 170 (19 June) because heavy precipitation occurred after the first treatment. Further applications were conducted on DOY 187 (6 July; bunch closure), DOY 192 (11 July; following precipitation), and at veraison, on DOY 201 (20 July) for *Assyrtiko* and DOY 203 (22 July) for Agiorgitiko. In 2023, applications were performed on DOY 158 (7 June), DOY 171 (20 June), and DOY 185 (4 July). Application timing followed the manufacturer’s recommendations and was adjusted according to phenological stage and rainfall events.

The vineyard was equipped with an in-line drip irrigation system, with one dripper per grapevine. In the fully irrigated treatment, each dripper supplied 8 L h^−1^, whereas in the deficit-irrigated treatments each dripper supplied 4 L h^−1^. Rainfed grapevines received no irrigation. The total water received by each treatment during the June–August period is presented in Table 6.

### 4.4. Leaf Area and Vegetative Growth

Vegetative growth was assessed only in 2022, due to the logistical demands of coordinating repeated field campaigns across five treatments and two cultivars within a single season. On 28 September, three representative shoots were collected from each grapevine, and five grapevines were sampled per treatment plot within each experimental block for each cultivar. Internode diameter was measured at the fifth node of each shoot using a digital caliper, and total shoot length was recorded with a measuring tape. Primary and secondary leaf areas were determined separately using a calibrated LI-3100C Area Meter (LI-COR, Inc., Lincoln, NE, USA). Leaves were placed individually on the conveyor belt to prevent overlap and minimize measurement error. For statistical analysis, measurements from the sampled shoots and grapevines within each treatment plot were averaged prior to analysis, and the treatment plot within each block was considered the biological replicate.

### 4.5. Gas Exchange and Leaf Temperature Measurements

Gas exchange and leaf temperature measurements were performed only during the 2022 growing season, owing to the logistical demands of repeated field campaigns with the portable photosynthesis system and infrared thermometer across five treatments and two cultivars. Leaf gas exchange was measured on well-exposed, fully expanded primary leaves located near the first cluster, generally between nodes 3 and 6. Measurements were conducted in the morning, between 09:00 and 10:00, and at midday, between 13:00 and 14:00, under stable weather conditions.

At each sampling date, gas-exchange measurements were obtained from representative tagged grapevines within each treatment plot and experimental block. For statistical analysis, one plot-level observation per treatment and block was used for each measurement date, with the treatment plot within each of the three blocks considered the biological replicate (*n* = 3). The same experimental plots were followed throughout the measurement period. Measurements were conducted on DOY 160 (9 June; pea-size berries), DOY 171 (20 June), DOY 178 (27 June), DOY 185 (4 July; bunch closure), DOY 203 (22 July; veraison for Assyrtiko), and DOY 206 (25 July; veraison for Agiorgitiko) using an LCpro+ portable photosynthesis system (ADC Bioscientific Ltd., Hoddesdon, UK). Measurements were recorded after gas exchange parameters had reached steady-state conditions. Leaf assimilation rate (A), transpiration rate (E), and stomatal conductance (g_s_) were determined, and intrinsic water use efficiency (WUEi) was calculated as the ratio of A to g_s_. During measurements, the leaf chamber was operated at a photosynthetically active radiation (PAR) of 1600 μmol m^−2^ s^−1^, a reference CO_2_ concentration of 400 μmol mol^−1^, a flow rate of 500 μmol s^−1^, and a leaf chamber area of 6.25 cm^2^.

Leaf temperature (Tleaf) was measured on tagged grapevines within the same experimental plots using an FLIR TG165-X infrared thermometer (FLIR Systems, Wilsonville, OR, USA). Measurements were performed on DOY 160, 171, 178, 185, 203, 206, and 242. Measurements on DOY 203 corresponded to veraison for Assyrtiko, whereas those on DOY 206 corresponded to veraison for Agiorgitiko. On DOY 242, leaf temperature was measured in both cultivars.

### 4.6. Yield Components

Yield components and berry composition were assessed in both the 2022 and 2023 growing seasons. Harvest was carried out at commercial maturity in each year. Assyrtiko was harvested on DOY 243 in 2022 and DOY 255 in 2023, corresponding to 31 August 2022 and 12 September 2023, respectively. Agiorgitiko was harvested on DOY 245 in 2022 and DOY 242 in 2023, corresponding to 2 September 2022 and 30 August 2023, respectively.

At harvest, the total number of clusters per grapevine was recorded, and all clusters were hand-harvested. Yield per grapevine was determined using a portable field scale (KERN & Sohn GmbH, CH15K20, Balingen, Germany). For cluster morphology, ten representative clusters were randomly sampled from each treatment plot within each experimental block and cultivar. Clusters were selected from primary shoots located in different parts of the canopy, including both shaded and exposed positions. Cluster length and width were measured using a digital caliper (as used for internode diameter measurements, Section 4.4), and cluster weight was determined using a precision electronic balance (PCB250-3, KERN & SOHN GmbH, Balingen, Germany).

For berry morphology, fifteen healthy berries were randomly collected from the sampled clusters within each treatment plot. Berry length and width were measured using a digital caliper, and fresh weight was determined using a precision electronic balance (PCB250-3, KERN & SOHN GmbH, Balingen, Germany, with a readability of 0.001 g), after which skins and seeds were separated and weighed individually on the same balance. Relative skin and seed mass were calculated as the percentage of total berry fresh weight [Relative skin mass (%) = (skin fresh weight/total berry fresh weight) × 100; relative seed mass (%) = (seed fresh weight/total berry fresh weight) × 100]. Measurements obtained from clusters and berries within each treatment plot were averaged prior to statistical analysis; therefore, the treatment plot within each block was considered the biological replicate.

### 4.7. Berry Composition and Quality Traits

Immediately after harvest, must samples from each treatment were analyzed for total soluble solids (TSS), pH, and titratable acidity (TA). TSS was measured at 20 °C using a digital handheld refractometer (PAL, Atago Co., Ltd., Tokyo, Japan) and expressed as °Brix. Must pH was measured using a digital pH meter (Hanna Instruments, Woonsocket, RI, USA). Titratable acidity was determined by titration with 0.1 N NaOH using bromothymol blue as an indicator, and results were expressed as g L^−1^ tartaric acid equivalents.

Total phenolic content (TPC) and total anthocyanin content were determined in berry skins after storage at −20 °C, according to the methods described by Slinkard and Singleton [47] and Singleton and Esau [48], respectively. Absorbance was measured using a UV–vis spectrophotometer (Optizen Pop, Mecacys, Daejeon, Republic of Korea) at 765 nm for TPC and 520 nm for total anthocyanins. Results were expressed as mg g^−1^ skin fresh weight.

As noted above, vegetative growth, gas exchange, and leaf temperature were assessed only in 2022 due to the logistical demands of repeated field campaigns with the portable photosynthesis system and infrared thermometry across five treatments and two cultivars. Yield and berry composition, which required a single end-of-season assessment, were evaluated in both 2022 and 2023.

### 4.8. Statistical Analysis

Statistical analyses were performed separately for each cultivar, using the treatment plot within block as the experimental unit (*n* = 3 plots per treatment). Measurements obtained from multiple shoots, grapevines, clusters, berries, or leaves within a treatment plot were averaged before inferential analysis so that subsamples were not treated as independent biological replicates. The fully irrigated untreated treatment (C) was retained as a non-stressed reference, while the four water-limited treatments (DI-NY, DI-Y, NI-NY, and NI-Y) constituted a 2 × 2 factorial subset with water condition (deficit irrigation vs. rainfed) and yeast application (untreated vs. yeast-treated) as factors.

For variables measured in a single season, including vegetative growth, the water-limited subset was analyzed using a randomized complete block linear model including Water, Yeast, Water × Yeast, and Block. For yield components and berry-composition variables measured in both 2022 and 2023, combined repeated models were fitted with treatment plot as the repeated subject and with Water, Yeast, Year, and all corresponding two- and three-way interactions as fixed effects. Gaussian generalized estimating equations with an exchangeable within-plot correlation structure and bias-reduced robust covariance estimation were used for these two-year analyses.

Gas-exchange and leaf-temperature data were analyzed as repeated measurements, separately for each cultivar and time of day. Plot-level means were the repeated observations, Date was treated as a categorical within-plot factor, and models included Water, Yeast, Date, and all interactions. Gaussian generalized estimating equations with an exchangeable within-plot correlation structure and bias-reduced robust covariance estimation were used. The C treatment was not included in these factorial interaction tests but was retained for graphical presentation and as a non-stressed reference.

To compare the yeast-treated water-limited treatments with the fully irrigated reference, separate five-level treatment models and selected planned contrasts (DI-Y − C and NI-Y − C) were used. Contrasts are reported as estimates with 95% confidence intervals and *p*-values and were not interpreted as equivalence or non-inferiority tests. For single-season linear models, residual normality and homogeneity of variance were evaluated using the Shapiro–Wilk and Levene tests, respectively. Statistical significance was accepted at *p* ≤ 0.05.

## 5. Conclusions

Water limitation affected vegetative growth, physiological performance, yield components, and berry composition of Agiorgitiko and Assyrtiko grapevines, but the response varied substantially between cultivars, traits, and seasons. The factorial analysis showed that water condition-dependent responses to the proline-rich yeast derivative were not universal. In Agiorgitiko, significant water × yeast interactions were detected for primary leaf area, shoot length, and all four morning gas-exchange variables, whereas in Assyrtiko such interactions were more evident for TSS and selected berry morphology traits.

Total yield did not show a significant water × yeast interaction in either cultivar, despite numerical increases in yeast-treated rainfed grapevines relative to untreated rainfed grapevines. Therefore, the observed yield improvement under rainfed conditions should be regarded as a descriptive treatment response rather than proof of greater yield mitigation by yeast under stronger water limitation. Leaf temperature likewise showed yeast- and date-dependent responses but no consistent water × yeast interaction.

Berry composition responses were particularly cultivar-specific. In Agiorgitiko, yeast application affected TSS, TA, and total phenolic content, with several responses varying between years but little evidence of an overall water × yeast interaction. In Assyrtiko, TSS showed a strong and consistent water × yeast interaction across both seasons, while TA, pH, and phenolic content were characterized mainly by year-dependent interactions. These findings demonstrate that physiological, productivity, and composition responses to the yeast derivative should be interpreted separately rather than summarized as a single uniform biostimulant effect.

Overall, foliar application of the proline-rich yeast derivative may contribute to grapevine adaptation under water-limited Mediterranean conditions, but its effectiveness is context-dependent. The present water-limited factorial analysis identifies specific cultivar–trait combinations in which the response to yeast application depends on the degree of water limitation, while also showing that this interaction does not extend to all outcomes. Future multi-site studies including a fully irrigated + yeast treatment and direct indicators of grapevine water status would help extend these findings across the complete irrigation gradient and refine practical application strategies.

## Figures and Tables

**Table 1:** Vegetative growth of Agiorgitiko and Assyrtiko grapevines under the five field treatments in 2022. Values are means ± SE of three treatment plots. Factorial *p*-values are from a two-way RCBD analysis restricted to DI-NY, DI-Y, NI-NY and NI-Y. C was excluded from the factorial model and retained as a non-stressed reference; selected contrasts versus C are reported in Table S2. No nonsignificant contrast is interpreted as evidence of equivalence.

	Treatments	Primary Leaf Area (cm^2^)	Secondary Leaf Area (cm^2^)	Internode Diameter (mm)	Shoot Length (cm)
Agiorgitiko	C	1396 ± 48	774 ± 97	7.23 ± 0.59	88.8 ± 4.3
	DI-NY	1337 ± 82	887 ± 69	6.06 ± 0.55	83.3 ± 6.3
	DI-Y	1494 ± 57	2159 ± 607	6.39 ± 1.19	85.4 ± 3.7
	NI-NY	800 ± 66	476 ± 92	4.80 ± 0.43	54.9 ± 6.8
	NI-Y	1623 ± 47	1131 ± 267	6.20 ± 0.37	102.2 ± 7.7
	Factorial *p*-values (water-limited subset)				
	Water regime (W)	0.021	0.103	0.333	0.067
	Yeast (Y)	<0.001	0.042	0.256	<0.001
	W × Y	0.002	0.442	0.465	<0.001
Assyrtiko	C	2018 ± 4	700 ± 200	7.17 ± 0.81	146.7 ± 24.4
	DI-NY	1629 ± 221	1135 ± 16	7.03 ± 0.86	141.0 ± 26.6
	DI-Y	1816 ± 106	1293 ± 13	7.00 ± 0.18	145.8 ± 8.4
	NI-NY	938 ± 21	662 ± 64	5.22 ± 0.18	101.3 ± 4.5
	NI-Y	1506 ± 23	965 ± 438	5.77 ± 1.08	134.8 ± 15.9
	Factorial *p*-values (water-limited subset)				
	Water regime (W)	0.003	0.112	0.110	0.081
	Yeast (Y)	0.011	0.325	0.761	0.165
	W × Y	0.115	0.749	0.731	0.281

**Table 2:** Yield components and cluster and berry traits of field-grown Agiorgitiko grapevines in 2022 and 2023. Values are means ± SE of three treatment plots. Factorial *p*-values are from the combined repeated two-year model for DI-NY, DI-Y, NI-NY, and NI-Y, including Water, Yeast, Year, and their interactions. C was excluded from the factorial model and retained as a non-stressed reference; selected contrasts versus C are re-ported in Table S2. NA, not assessed in 2023.

	Treatments	Yield (kg/Vine)	Clusters/ Vine	Cluster Weight (g)	Cluster Length (cm)	Cluster Width (cm)	Cluster Compactness (g/cm)	Berry Weight (g)	Berry Length (mm)	Berry Width (mm)	Relative Skin Mass (%)	Relative Seed Mass (%)
2022	C	3.77 ± 0.19	16.67 ± 0.33	230.38 ± 12.30	14.08 ± 0.08	8.42 ± 0.96	16.38 ± 0.88	2.20 ± 0.07	15.98 ± 0.12	14.48 ± 0.45	10.63 ± 0.44	3.17 ± 0.11
	DI-NY	3.81 ± 1.12	19.00 ± 0.00	172.55 ± 15.12	14.00 ± 1.13	9.83 ± 1.09	12.41 ± 0.99	1.97 ± 0.11	14.22 ± 0.33	12.89 ± 0.31	11.61 ± 0.88	3.24 ± 0.19
	DI-Y	3.15 ± 0.49	18.67 ± 2.19	169.23 ± 19.15	13.17 ± 0.25	8.11 ± 0.70	12.85 ± 1.50	1.92 ± 0.02	14.42 ± 0.16	12.88 ± 0.92	9.59 ± 0.32	3.19 ± 0.05
	NI-NY	2.77 ± 0.40	18.00 ± 3.00	154.43 ± 13.50	12.83 ± 1.26	8.83 ± 0.58	12.30 ± 0.87	1.78 ± 0.06	14.13 ± 0.66	12.80 ± 0.52	10.58 ± 0.58	3.76 ± 0.53
	NI-Y	3.00 ± 0.41	19.33 ± 2.19	158.32 ± 16.22	13.00 ± 0.52	9.08 ± 0.08	12.35 ± 1.36	2.02 ± 0.20	14.35 ± 0.25	13.86 ± 0.52	11.48 ± 0.59	3.49 ± 0.14
2023	C	2.77 ± 0.17	16.50 ± 1.04	168.98 ± 5.37	14.97 ± 0.12	8.50 ± 0.29	11.32 ± 0.38	1.50 ± 0.10	NA	NA	NA	NA
	DI-NY	2.26 ± 0.68	16.33 ± 4.91	135.50 ± 28.39	15.89 ± 0.39	8.92 ± 1.21	8.56 ± 1.78	1.25 ± 0.12	NA	NA	NA	NA
	DI-Y	2.30 ± 0.12	16.67 ± 0.88	136.05 ± 13.32	14.28 ± 0.53	8.11 ± 0.36	9.42 ± 0.81	1.74 ± 0.33	NA	NA	NA	NA
	NI-NY	1.19 ± 0.13	13.33 ± 1.42	89.27 ± 17.94	12.00 ± 0.88	7.25 ± 0.63	7.68 ± 1.71	1.24 ± 0.06	NA	NA	NA	NA
	NI-Y	1.80 ± 0.33	14.33 ± 2.59	125.77 ± 1.98	14.08 ± 0.94	8.28 ± 0.09	9.23 ± 0.54	1.09 ± 0.16	NA	NA	NA	NA
Factorial *p*-values (water-limited subset)												
Water regime (W)		0.102	0.577	0.380	0.011	0.287	0.792	0.264	NA	NA	NA	NA
Yeast (Y)		0.892	0.818	0.700	0.927	0.384	0.652	0.429	NA	NA	NA	NA
W × Y		0.387	0.818	0.658	0.027	0.008	0.962	0.610	NA	NA	NA	NA
Year		0.027	0.107	<0.001	0.359	0.306	<0.001	<0.001	NA	NA	NA	NA
W × Year		0.875	0.574	0.314	0.438	0.649	0.900	0.251	NA	NA	NA	NA
Y × Year		0.643	0.970	0.181	0.748	0.599	0.592	0.779	NA	NA	NA	NA
W × Y × Year		0.889	0.910	0.292	0.447	0.966	0.761	0.060	NA	NA	NA	NA

**Table 3:** Yield components and cluster and berry traits of field-grown Assyrtiko grapevines in 2022 and 2023. Values are means ± SE of three treatment plots. Factorial *p*-values are from the combined repeated two-year model for DI-NY, DI-Y, NI-NY, and NI-Y, including Water, Yeast, Year, and their interactions. C was excluded from the factorial model and retained as a non-stressed reference; selected contrasts versus C are reported in Table S2.

	Treatments	Yield (kg/vine)	Clusters/vine	Cluster Weight (g)	Cluster Length (cm)	Cluster Width (cm)	Cluster Compactness (g/cm)	Berry Weight (g)	Berry Length (mm)	Berry Width (mm)	Relative Skin Mass (%)	Relative Seed Mass (%)
2022	C	3.57 ± 0.26	17.17 ± 0.60	196.31 ± 24.22	16.59 ± 0.69	9.17 ± 1.00	12.14 ± 0.60	2.80 ± 0.05	16.50 ± 0.08	14.87 ± 0.06	12.62 ± 0.62	4.72 ± 0.78
	DI-NY	2.40 ± 0.10	15.17 ± 0.44	163.95 ± 19.36	15.78 ± 0.55	10.92 ± 0.17	10.39 ± 1.16	1.84 ± 0.02	14.70 ± 0.27	13.30 ± 0.15	9.07 ± 0.56	6.17 ± 0.20
	DI-Y	2.38 ± 0.25	11.83 ± 0.73	196.31 ± 24.22	15.33 ± 0.83	9.83 ± 0.65	12.73 ± 1.50	3.12 ± 0.25	18.16 ± 0.64	16.71 ± 0.35	9.60 ± 0.60	3.82 ± 0.13
	NI-NY	2.10 ± 0.05	13.33 ± 0.33	158.19 ± 18.08	16.28 ± 0.89	9.36 ± 0.74	9.44 ± 1.10	1.68 ± 0.15	14.07 ± 0.42	12.91 ± 0.31	8.42 ± 0.85	4.68 ± 0.42
	NI-Y	3.24 ± 0.46	16.50 ± 1.32	197.51 ± 26.87	16.25 ± 1.25	9.00 ± 0.88	11.94 ± 0.17	1.99 ± 0.12	14.85 ± 0.28	13.69 ± 0.54	7.22 ± 0.35	4.74 ± 0.41
2023	C	2.05 ± 0.51	17.67 ± 2.19	137.50 ± 10.64	16.72 ± 1.68	8.00 ± 0.38	8.24 ± 0.42	1.86 ± 0.09	14.01 ± 0.10	13.41 ± 0.36	13.57 ± 0.60	4.07 ± 0.18
	DI-NY	2.06 ± 0.53	18.00 ± 3.51	112.58 ± 15.16	15.44 ± 0.71	8.00 ± 0.29	7.25 ± 0.61	1.84 ± 0.04	14.50 ± 0.42	12.49 ± 0.17	11.19 ± 0.86	3.67 ± 0.09
	DI-Y	2.06 ± 0.19	17.33 ± 1.20	118.27 ± 15.09	14.94 ± 0.29	6.39 ± 0.20	3.65 ± 0.12	1.75 ± 0.07	13.85 ± 0.37	12.51 ± 0.20	12.85 ± 0.37	3.61 ± 0.05
	NI-NY	1.59 ± 0.67	17.00 ± 4.36	93.74 ± 12.74	15.33 ± 0.44	7.33 ± 0.60	6.08 ± 0.72	1.36 ± 0.02	14.69 ± 0.06	12.87 ± 0.06	10.93 ± 1.09	4.22 ± 0.11
	NI-Y	2.13 ± 0.16	19.67 ± 1.33	110.11 ± 11.05	16.36 ± 0.31	7.56 ± 0.56	6.57 ± 0.53	1.41 ± 0.04	12.36 ± 0.29	10.91 ± 0.16	12.08 ± 0.84	5.66 ± 0.10
Factorial *p*-values (water-limited subset)												
Water regime (W)		0.927	0.663	0.724	0.235	0.500	1.000	<0.001	<0.001	<0.001	0.138	0.034
Yeast (Y)		0.329	0.848	0.294	0.981	0.311	0.640	0.001	0.406	0.055	0.433	0.345
W × Y		0.314	0.304	0.843	0.397	0.361	0.252	0.077	0.004	<0.001	0.413	<0.001
Year		0.057	0.030	<0.001	0.549	<0.001	<0.001	<0.001	<0.001	<0.001	<0.001	<0.001
W × Year		0.420	0.830	0.713	0.966	0.109	0.249	0.212	0.044	0.029	0.483	<0.001
Y × Year		0.632	0.757	0.416	0.700	0.975	0.009	<0.001	<0.001	<0.001	0.220	<0.001
W × Y × Year		0.609	0.651	0.951	0.669	0.537	0.196	0.003	0.449	0.508	0.669	0.157

**Table 4:** Berry composition of field-grown Agiorgitiko grapevines under the five field treatments in 2022 and 2023. Values are means ± SE of three treatment plots. Factorial *p*-values are from the combined repeated two-year model for DI-NY, DI-Y, NI-NY, and NI-Y, including Water, Yeast, Year, and their interactions. C was retained as a non-stressed reference and was not included in the factorial interaction test; selected contrasts versus C are reported in Table S2.

	Treatments	TSS (°Brix)	TA (g Tartaric Acid/L)	pH	Total Phenolic Content (mg/g Skin Fresh Weight)	Total Anthocyanin Content (mg/g Skin Fresh Weight)
2022	C	24.09 ± 0.16	4.86 ± 0.10	3.37 ± 0.04	1.97 ± 0.16	0.98 ± 0.23
	DI-NY	25.07 ± 0.32	4.70 ± 0.03	3.42 ± 0.00	2.21 ± 0.05	1.15 ± 0.06
	DI-Y	23.06 ± 0.37	5.21 ± 0.02	3.28 ± 0.02	2.58 ± 0.09	1.20 ± 0.06
	NI-NY	24.52 ± 0.08	4.83 ± 0.09	3.36 ± 0.03	2.25 ± 0.27	1.23 ± 0.09
	NI-Y	23.40 ± 0.13	4.70 ± 0.05	3.41 ± 0.01	2.17 ± 0.04	1.20 ± 0.00
2023	C	25.98 ± 1.49	6.15 ± 0.68	3.61 ± 0.02	2.23 ± 0.12	0.90 ± 0.06
	DI-NY	27.52 ± 1.98	5.67 ± 0.16	3.60 ± 0.05	2.17 ± 0.07	0.95 ± 0.02
	DI-Y	26.68 ± 1.03	6.50 ± 0.39	3.75 ± 0.01	2.18 ± 0.07	1.07 ± 0.08
	NI-NY	27.25 ± 0.56	5.40 ± 0.23	3.61 ± 0.04	2.07 ± 0.19	1.17 ± 0.11
	NI-Y	24.22 ± 1.17	6.70 ± 0.33	3.65 ± 0.01	2.33 ± 0.07	1.17 ± 0.06
Factorial *p*-values (water-limited subset)						
Water regime (W)		0.307	0.519	0.817	0.212	0.055
Yeast (Y)		0.015	<0.001	0.293	0.033	0.485
W × Y		0.648	0.821	0.364	0.441	0.309
Year		0.001	<0.001	<0.001	0.443	0.157
W × Year		0.390	0.644	0.144	0.481	0.407
Y × Year		0.802	0.010	0.011	0.980	0.755
W × Y × Year		0.294	0.100	0.008	0.245	0.896

**Table 5:** Berry composition of field-grown Assyrtiko grapevines under the five field treatments in 2022 and 2023. Values are means ± SE of three treatment plots. Factorial *p*-values are from the combined repeated two-year model for DI-NY, DI-Y, NI-NY, and NI-Y, including Water, Yeast, Year, and their interactions. C was retained as a non-stressed reference and was not included in the factorial interaction test; selected contrasts versus C are reported in Table S2.

	Treatments	TSS (°Brix)	TA (g Tartaric Acid/L)	pH	Total Phenolic Content (mg/g Skin Fresh Weight)
2022	C	20.61 ± 0.28	5.54 ± 0.05	3.36 ± 0.07	0.96 ± 0.00
	DI-NY	20.31 ± 0.16	6.95 ± 0.11	3.23 ± 0.05	1.15 ± 0.01
	DI-Y	20.29 ± 0.45	5.20 ± 0.07	3.42 ± 0.07	1.33 ± 0.06
	NI-NY	22.50 ± 0.28	6.30 ± 0.16	3.39 ± 0.09	0.80 ± 0.13
	NI-Y	20.32 ± 0.06	6.15 ± 0.04	3.41 ± 0.12	1.49 ± 0.21
2023	C	18.92 ± 0.03	5.70 ± 0.11	3.59 ± 0.01	0.96 ± 0.02
	DI-NY	18.75 ± 0.00	6.58 ± 0.03	3.48 ± 0.00	1.07 ± 0.00
	DI-Y	18.62 ± 0.28	7.77 ± 0.31	3.25 ± 0.00	1.33 ± 0.06
	NI-NY	20.65 ± 0.00	7.12 ± 0.15	3.35 ± 0.02	0.80 ± 0.13
	NI-Y	18.62 ± 0.28	7.77 ± 0.31	3.25 ± 0.00	1.49 ± 0.21
Factorial *p*-values (water-limited subset)					
Water regime (W)		<0.001	0.255	0.948	0.722
Yeast (Y)		<0.001	0.947	0.702	0.028
W × Y		<0.001	0.159	0.930	0.246
Year		<0.001	<0.001	0.571	<0.001
W × Year		0.761	0.691	0.195	<0.001
Y × Year		0.973	<0.001	0.015	<0.001
W × Y × Year		0.806	<0.001	0.185	<0.001

**Table 6:** Total water input, including rainfall and irrigation, recorded from June to August during the 2022 and 2023 growing seasons in field-grown Agiorgitiko and Assyrtiko grapevines subjected to different water regimes and foliar application of LalVigne ProHydro™. C = fully irrigated untreated grapevines; DI-NY and DI-Y = deficit-irrigated grapevines without and with yeast application, respectively; NI-NY and NI-Y = rainfed grapevines without and with yeast application, respectively. Values represent total water input per treatment across the June–August period; *n* = 3 blocks.

Treatments	2022	2023
C	173.5 mm	356.5 mm
DI (NY, Y)	150.7 mm	192.5 mm
NI (NY, Y)	127.9 mm	28.5 mm

## Data Availability

Data will be made available on request.

## Supplementary Materials

The following supporting information can be downloaded at https://www.mdpi.com/article/10.3390/plants15172631/s1: Figure S1. Infrared thermal images of Agiorgitiko leaves opposite the first cluster, recorded in July 2022 under the five field treatments; Figure S2. Infrared thermal images of Assyrtiko leaves opposite the first cluster, recorded in July 2022 under the five field treatments; Figure S3. Representative Agiorgitiko clusters at harvest in 2022 under the five field treatments; Figure S4. Representative Assyrtiko clusters at harvest in 2022 under the five field treatments; Figure S5. Agiorgitiko clusters sampled for chemical and morphological analyses in 2022 under the five field treatments; Figure S6. Assyrtiko clusters sampled for chemical and morphological analyses in 2022 under the five field treatments; Figure S7. Seasonal evolution of soil volumetric water content (VWC, %) under the three irrigation regimes in Agiorgitiko and Assyrtiko grapevines during the 2022 growing season; Figure S8. Seasonal evolution of soil volumetric water content (VWC, %) under the three irrigation regimes in Agiorgitiko and Assyrtiko grapevines during the 2023 growing season. Table S1. Repeated-measures factorial effects for gas exchange and leaf temperature in 2022; Table S2. Selected planned contrasts of the yeast-treated water-limited treatments (DI-Y and NI-Y) versus the fully irrigated untreated reference (C).
