# Supplementary material for: Mitigating Water-Deficit Stress in Mediterranean Grapevines: Field Responses of Agiorgitiko and Assyrtiko to a Proline-Rich Yeast Derivative
Source: Plants (Basel). 2026 Aug 28;15(17):2631. doi: 10.3390/plants15172631 (PMC13567535; doi:10.3390/plants15172631)
Supplement: Supplementary file 1 [file plants-15-02631-s001.zip › plants-4483460-supplementary.pdf]

Mitigating Water-Deficit Stress in Mediterranean Grapevines: Field Responses of Agiorgitiko and Assyrtiko to a Proline-Rich Yeast Derivative

Despoina G. Petoumenou[1], Athanasia Tsigkreli[1], Christos Spyridon Dimpertis[1], Dimitrios Saripavlidis[1], Emmanouil Kontaxakis[2] and Spyridon Souipas[1]

[1] Laboratory of Viticulture, Department of Agriculture, Crop Production and Rural Environment, University of Thessaly, Fytokou Street, 384 46 Volos, Greece;

[2] Department of Agriculture, School of Agricultural Sciences, Hellenic Mediterranean University, P.O. Box 1939, GR 71410 Heraklion, Greece;

*Correspondence: petoumenou@uth.gr

Supplementary Materials - Figure S1

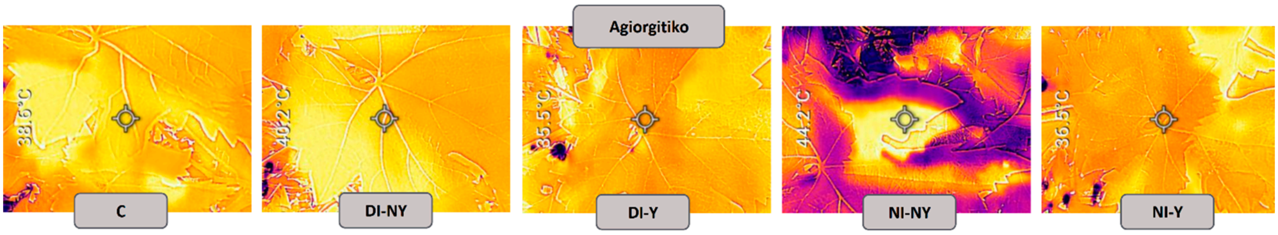

Figure S1. Infrared thermal images of Agiorgitiko leaves opposite the first cluster, recorded in July 2022 under the five field treatments. C = fully irrigated untreated grapevines; DI-NY = deficit-irrigated untreated grapevines; DI-Y = deficit-irrigated yeast-treated grapevines; NI-NY = rainfed untreated grapevines; NI-Y = rainfed yeast-treated grapevines.

Supplementary Materials - Figure S2

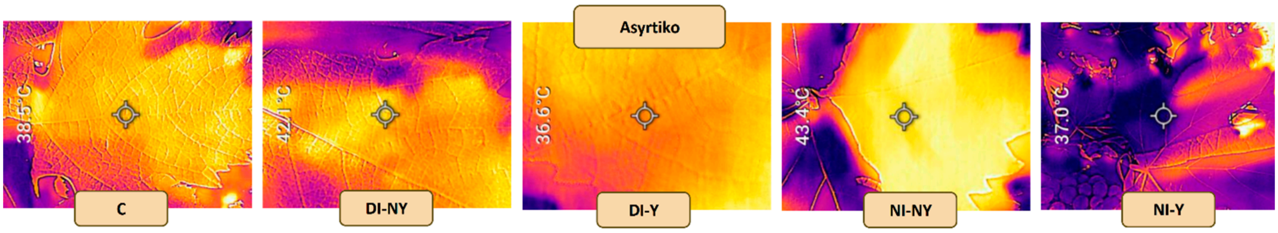

Figure S2. Infrared thermal images of Assyrtiko leaves opposite the first cluster, recorded in July 2022 under the five field treatments. C = fully irrigated untreated grapevines; DI-NY = deficit-irrigated untreated grapevines; DI-Y = deficit-irrigated yeast-treated grapevines; NI-NY = rainfed untreated grapevines; NI-Y = rainfed yeast-treated grapevines.

Supplementary Materials - Figure S3

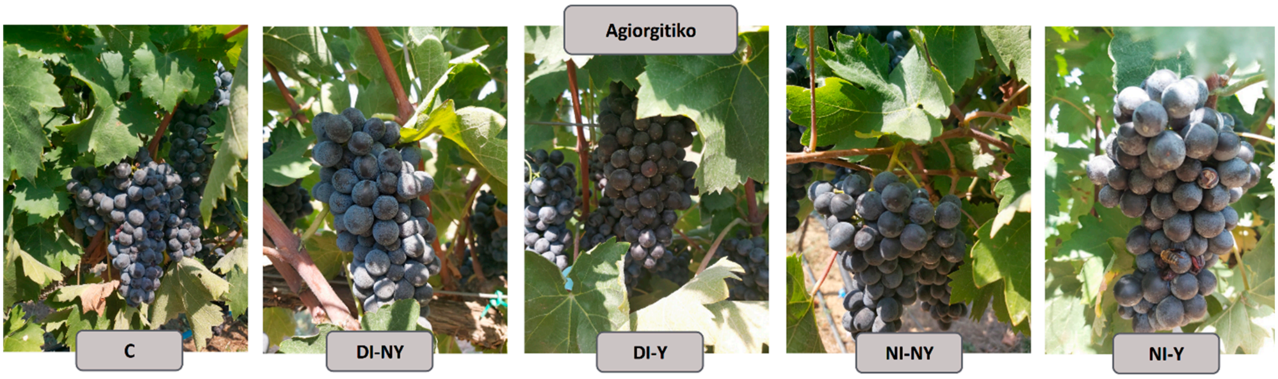

Figure S3. Representative clusters of Agiorgitiko grapevines at harvest in 2022 under the five field treatments. C = fully irrigated untreated grapevines; DI-NY = deficit-irrigated untreated grapevines; DI-Y = deficit-irrigated yeast-treated grapevines; NI-NY = rainfed untreated grapevines; NI-Y = rainfed yeast-treated grapevines.

Supplementary Materials - Figure S4

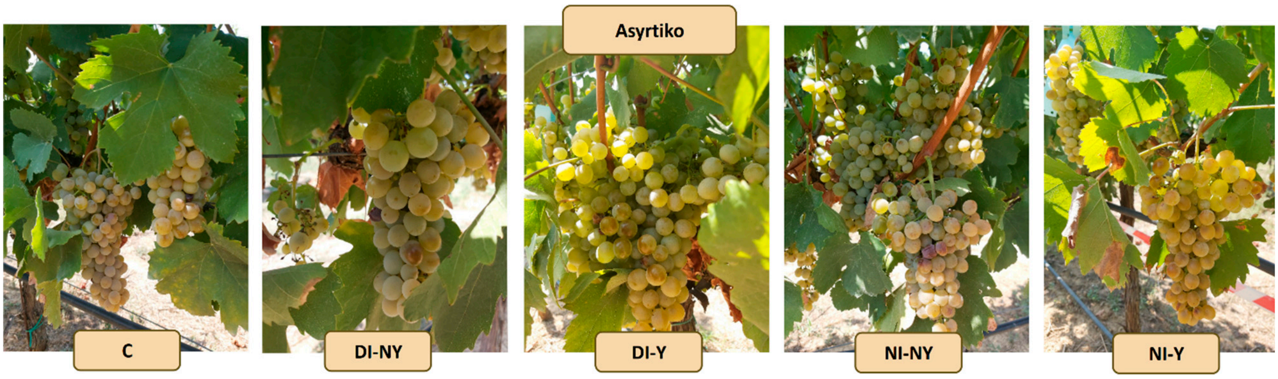

Figure S4. Representative clusters of Assyrtiko grapevines at harvest in 2022 under the five field treatments. C = fully irrigated untreated grapevines; DI-NY = deficit-irrigated untreated grapevines; DI-Y = deficit-irrigated yeast-treated grapevines; NI-NY = rainfed untreated grapevines; NI-Y = rainfed yeast-treated grapevines.

Supplementary Materials - Figure S5

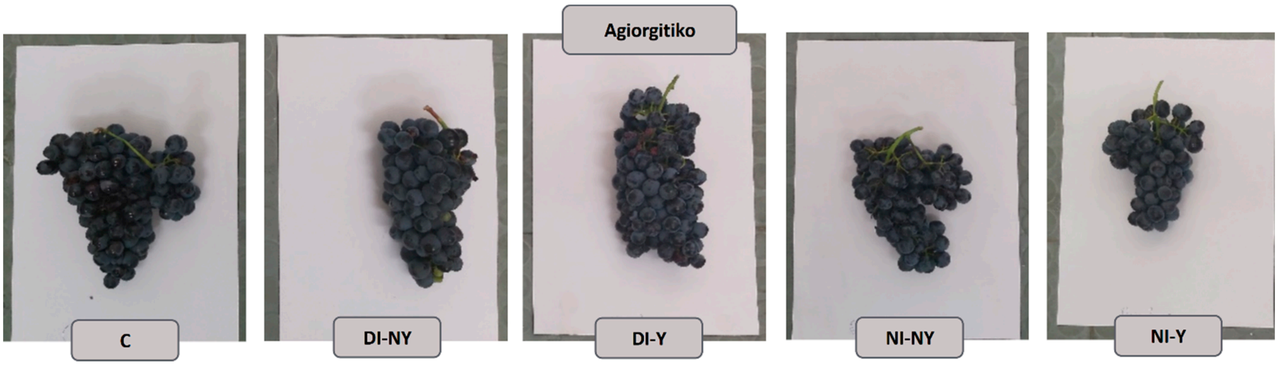

Figure S5. Agiorgitiko clusters sampled for chemical and morphological analyses in 2022 under the five field treatments. C = fully irrigated untreated grapevines; DI-NY = deficit-irrigated untreated grapevines; DI-Y = deficit-irrigated yeast-treated grapevines; NI-NY = rainfed untreated grapevines; NI-Y = rainfed yeast-treated grapevines.

Supplementary Materials - Figure S6

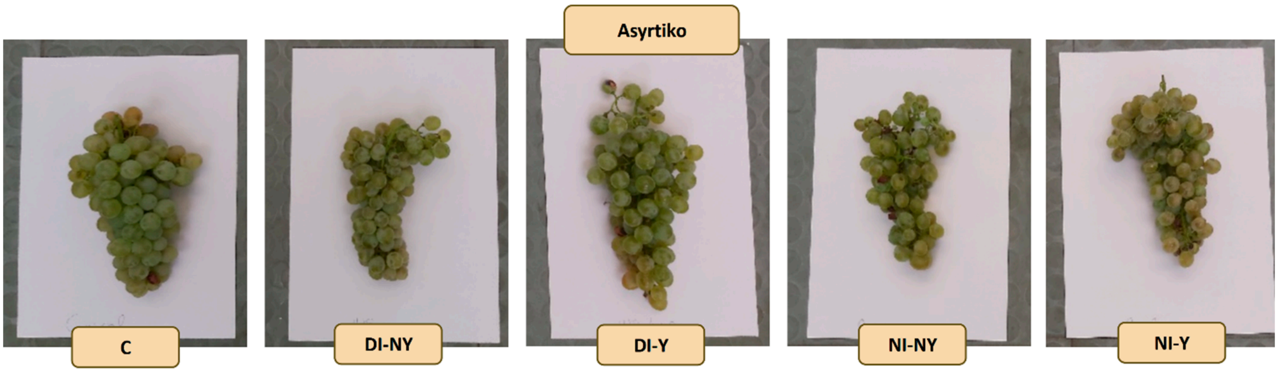

Figure S6. Assyrtiko clusters sampled for chemical and morphological analyses in 2022 under the five field treatments. C = fully irrigated untreated grapevines; DI-NY = deficit-irrigated untreated grapevines; DI-Y = deficit-irrigated yeast-treated grapevines; NI-NY = rainfed untreated grapevines; NI-Y = rainfed yeast-treated grapevines.

Supplementary Materials - Figure S7

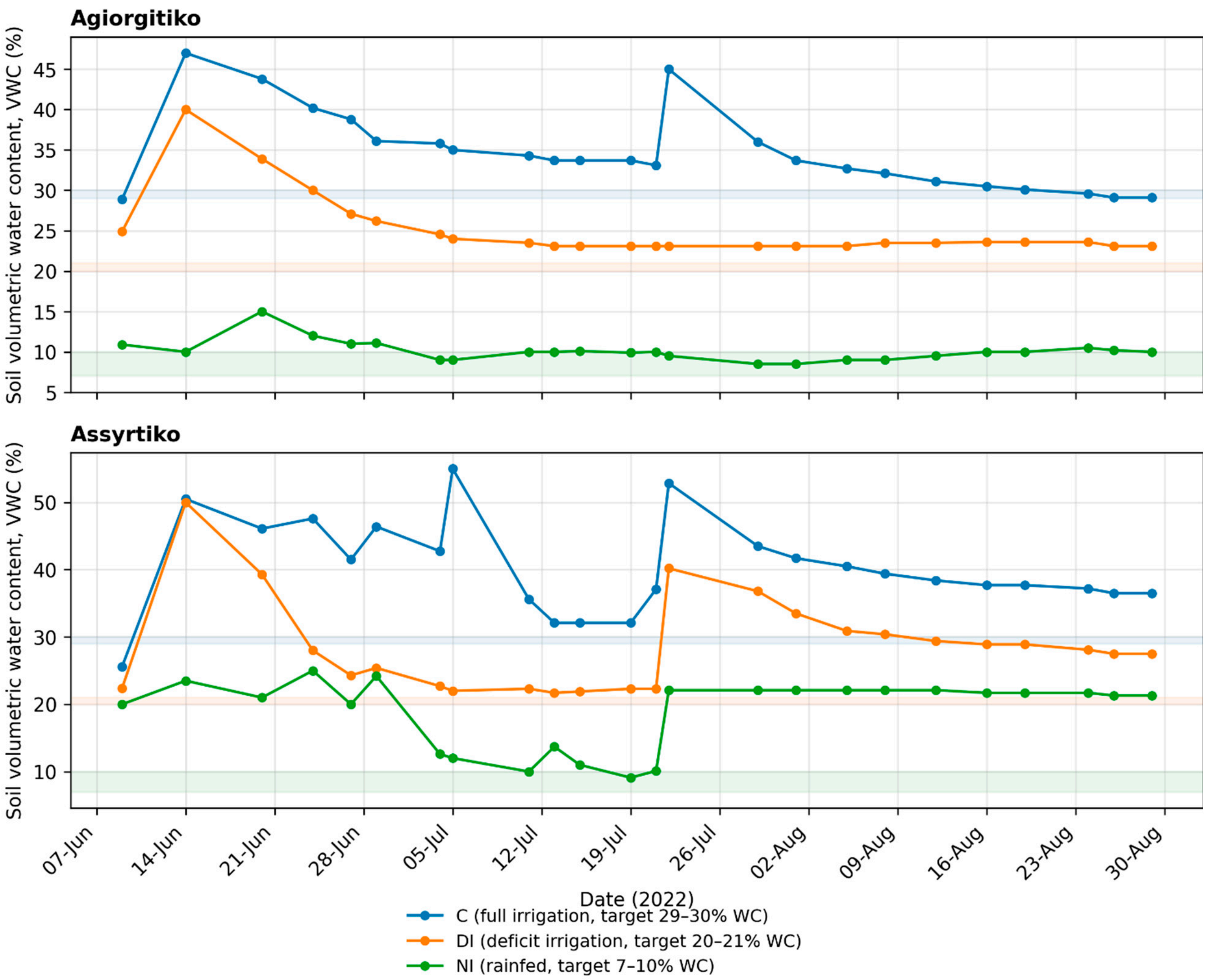

Figure S7. Seasonal evolution of soil volumetric water content (VWC, %) under the three irrigation regimes in Agiorgitiko and Assyrtiko grapevines during the 2022 growing season.

Supplementary Materials - Figure S8

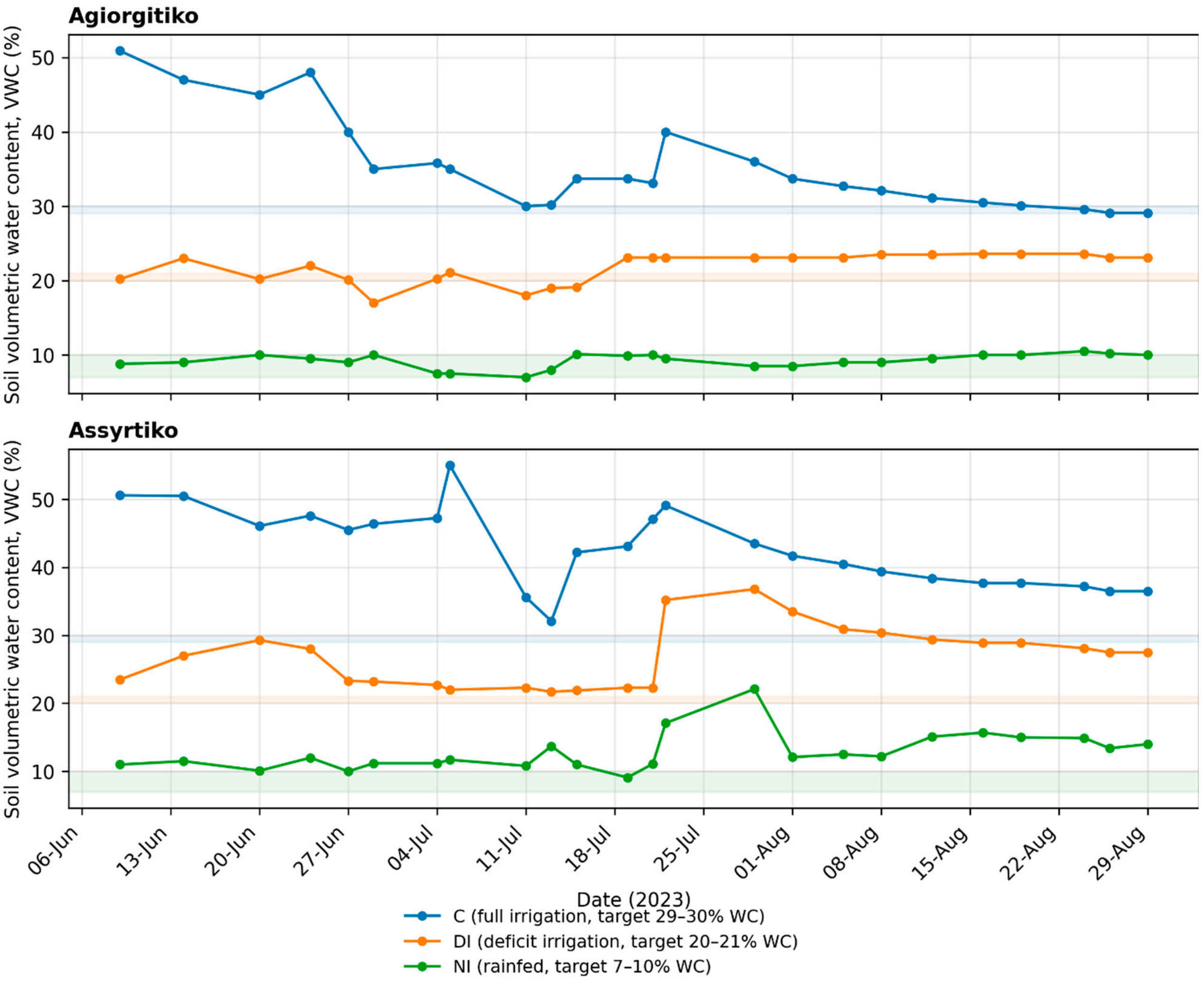

Figure S8. Seasonal evolution of soil volumetric water content (VWC, %) under the three irrigation regimes in Agiorgitiko and Assyrtiko grapevines during the 2023 growing season.

Supplementary Materials - Table S1

Table S1. Repeated-measures factorial effects for gas exchange and leaf temperature in 2022. Formal factorial inference was based on the four water-limited treatments (DI-NY, DI-Y, NI-NY, and NI-Y), with treatment plot as the repeated subject. The fully irrigated untreated treatment (C) was retained as a non-stressed reference and was not included in the factorial interaction tests.

Cultivar	Time	Variable	Water	Yeast	W × Y	Date	W × Date	Y × Date	W × Y × Date
Agiorgitiko	Morning	A	<0.001	<0.001	<0.001	<0.001	0.401	0.211	0.138
Agiorgitiko	Morning	E	<0.001	<0.001	0.012	<0.001	0.004	0.003	<0.001
Agiorgitiko	Morning	gs	<0.001	<0.001	0.015	<0.001	0.312	0.497	0.011
Agiorgitiko	Morning	WUEi	<0.001	<0.001	0.005	<0.001	<0.001	0.059	0.104
Agiorgitiko	Midday	A	0.020	<0.001	0.076	<0.001	<0.001	0.012	0.385
Agiorgitiko	Midday	E	0.131	<0.001	0.836	<0.001	0.479	0.419	0.181
Agiorgitiko	Midday	gs	0.035	0.002	0.306	0.031	0.042	0.198	0.438
Agiorgitiko	Midday	WUEi	0.493	0.376	0.986	<0.001	0.669	0.107	0.367
Assyrtiko	Morning	A	<0.001	<0.001	0.351	<0.001	0.178	0.002	0.250
Assyrtiko	Morning	E	0.039	<0.001	0.815	<0.001	0.382	0.039	0.009
Assyrtiko	Morning	gs	0.742	0.962	0.346	<0.001	0.586	0.055	0.034
Assyrtiko	Morning	WUEi	0.818	0.768	0.657	<0.001	0.353	0.108	0.067
Assyrtiko	Midday	A	<0.001	0.143	0.184	<0.001	0.045	<0.001	0.379
Assyrtiko	Midday	E	0.009	0.353	0.177	0.067	0.124	0.485	0.733
Assyrtiko	Midday	gs	0.080	0.833	0.233	0.002	0.174	0.402	0.520
Assyrtiko	Midday	WUEi	0.332	0.633	0.352	0.040	0.697	0.390	0.552
Agiorgitiko	Morning	Tleaf	0.262	<0.001	0.783	<0.001	<0.001	0.349	0.088
Agiorgitiko	Midday	Tleaf	0.293	0.021	0.344	<0.001	0.435	0.128	0.422
Assyrtiko	Morning	Tleaf	0.841	<0.001	0.808	<0.001	<0.001	<0.001	<0.001
Assyrtiko	Midday	Tleaf	0.943	0.543	0.535	<0.001	0.002	<0.001	0.484

Supplementary Materials - Table S2

Table S2. Selected planned contrasts of the yeast-treated water-limited treatments (DI-Y and NI-Y) versus the fully irrigated untreated reference (C). Values are treatment - C estimates with 95% confidence intervals and p-values. These contrasts assess differences from C and are not equivalence or non-inferiority tests.

Vegetative growth

Cultivar	Year / Time	Variable	Contrast	Estimate (95% CI)	p
Agiorgitiko	—	Primary leaf area	DI-Y - C	98.733 (-95.046, 292.513)	0.274
Agiorgitiko	—	Primary leaf area	NI-Y - C	227.133 (33.354, 420.913)	0.027
Agiorgitiko	—	Secondary leaf area	DI-Y - C	1385.050 (311.746, 2458.354)	0.018
Agiorgitiko	—	Secondary leaf area	NI-Y - C	357.100 (-716.204, 1430.404)	0.465
Agiorgitiko	—	Internode diameter	DI-Y - C	-0.847 (-2.821, 1.128)	0.352
Agiorgitiko	—	Internode diameter	NI-Y - C	-1.033 (-3.008, 0.941)	0.262
Agiorgitiko	—	Shoot length	DI-Y - C	-3.400 (-19.106, 12.306)	0.631
Agiorgitiko	—	Shoot length	NI-Y - C	13.333 (-2.373, 29.039)	0.086
Assyrtiko	—	Primary leaf area	DI-Y - C	-202.517 (-521.820, 116.787)	0.182
Assyrtiko	—	Primary leaf area	NI-Y - C	-512.200 (-831.503, -192.897)	0.006
Assyrtiko	—	Secondary leaf area	DI-Y - C	593.467 (-165.954, 1352.888)	0.109
Assyrtiko	—	Secondary leaf area	NI-Y - C	264.967 (-494.454, 1024.388)	0.444
Assyrtiko	—	Internode diameter	DI-Y - C	-0.170 (-2.768, 2.428)	0.884
Assyrtiko	—	Internode diameter	NI-Y - C	-1.400 (-3.998, 1.198)	0.249
Assyrtiko	—	Shoot length	DI-Y - C	-0.833 (-64.007, 62.341)	0.976
Assyrtiko	—	Shoot length	NI-Y - C	-11.833 (-75.007, 51.341)	0.677

Yield, cluster, and berry traits

Cultivar	Year / Time	Variable	Contrast	Estimate (95% CI)	p
Agiorgitiko	2022	Yield	DI-Y - C	-0.612 (-2.564, 1.341)	0.491
Agiorgitiko	2022	Yield	NI-Y - C	-0.763 (-2.716, 1.189)	0.394
Agiorgitiko	2023	Yield	DI-Y - C	-0.466 (-1.538, 0.606)	0.345
Agiorgitiko	2023	Yield	NI-Y - C	-0.966 (-2.038, 0.106)	0.071
Agiorgitiko	2022	Clusters/vine	DI-Y - C	2.000 (-4.612, 8.612)	0.505

Cultivar	Year / Time	Variable	Contrast	Estimate (95% CI)	p
Agiorgitiko	2022	Clusters/vine	NI-Y - C	2.667 (-3.946, 9.279)	0.380
Agiorgitiko	2023	Clusters/vine	DI-Y - C	0.167 (-8.071, 8.404)	0.964
Agiorgitiko	2023	Clusters/vine	NI-Y - C	-2.167 (-10.404, 6.071)	0.561
Agiorgitiko	2022	Cluster weight	DI-Y - C	-61.150 (-117.333, -4.967)	0.036
Agiorgitiko	2022	Cluster weight	NI-Y - C	-72.067 (-128.250, -15.884)	0.018
Agiorgitiko	2023	Cluster weight	DI-Y - C	-32.924 (-87.997, 22.148)	0.205
Agiorgitiko	2023	Cluster weight	NI-Y - C	-43.209 (-98.282, 11.864)	0.108
Agiorgitiko	2022	Cluster length	DI-Y - C	-0.917 (-3.787, 1.953)	0.482
Agiorgitiko	2022	Cluster length	NI-Y - C	-1.083 (-3.953, 1.787)	0.409
Agiorgitiko	2023	Cluster length	DI-Y - C	-0.694 (-2.468, 1.079)	0.393
Agiorgitiko	2023	Cluster length	NI-Y - C	-0.889 (-2.663, 0.885)	0.281
Agiorgitiko	2022	Cluster width	DI-Y - C	-0.306 (-2.532, 1.920)	0.760
Agiorgitiko	2022	Cluster width	NI-Y - C	0.667 (-1.559, 2.893)	0.509
Agiorgitiko	2023	Cluster width	DI-Y - C	-0.389 (-1.974, 1.196)	0.587
Agiorgitiko	2023	Cluster width	NI-Y - C	-0.220 (-1.805, 1.365)	0.757
Agiorgitiko	2022	Cluster compactness	DI-Y - C	-3.531 (-7.632, 0.570)	0.082
Agiorgitiko	2022	Cluster compactness	NI-Y - C	-4.035 (-8.136, 0.066)	0.053
Agiorgitiko	2023	Cluster compactness	DI-Y - C	-1.899 (-6.249, 2.451)	0.344
Agiorgitiko	2023	Cluster compactness	NI-Y - C	-2.088 (-6.438, 2.263)	0.301
Agiorgitiko	2022	Berry weight	DI-Y - C	-0.281 (-0.683, 0.121)	0.146
Agiorgitiko	2022	Berry weight	NI-Y - C	-0.176 (-0.578, 0.226)	0.343
Agiorgitiko	2023	Berry weight	DI-Y - C	0.241 (-0.187, 0.668)	0.231
Agiorgitiko	2023	Berry weight	NI-Y - C	-0.414 (-0.841, 0.014)	0.056
Agiorgitiko	2022	Berry length	DI-Y - C	-1.560 (-2.734, -0.386)	0.015
Agiorgitiko	2022	Berry length	NI-Y - C	-1.629 (-2.802, -0.455)	0.013
Agiorgitiko	2022	Berry width	DI-Y - C	-1.601 (-3.396, 0.194)	0.074
Agiorgitiko	2022	Berry width	NI-Y - C	-0.619 (-2.414, 1.176)	0.450
Agiorgitiko	2022	Relative skin mass	DI-Y - C	-1.042 (-2.569, 0.485)	0.154
Agiorgitiko	2022	Relative skin mass	NI-Y - C	0.850 (-0.677, 2.377)	0.235
Agiorgitiko	2022	Relative seed mass	DI-Y - C	0.015 (-0.926, 0.956)	0.971

Cultivar	Year / Time	Variable	Contrast	Estimate (95% CI)	p
Agiorgitiko	2022	Relative seed mass	NI-Y - C	0.318 (-0.623, 1.259)	0.458
Assyrtiko	2022	Yield	DI-Y - C	-1.191 (-1.994, -0.388)	0.009
Assyrtiko	2023	Yield	DI-Y - C	0.007 (-1.639, 1.652)	0.993
Assyrtiko	2023	Yield	NI-Y - C	0.073 (-1.572, 1.719)	0.921
Assyrtiko	2022	Clusters/vine	DI-Y - C	-5.333 (-7.900, -2.767)	0.001
Assyrtiko	2022	Clusters/vine	NI-Y - C	-0.667 (-3.233, 1.900)	0.566
Assyrtiko	2023	Clusters/vine	DI-Y - C	-0.333 (-10.338, 9.671)	0.941
Assyrtiko	2023	Clusters/vine	NI-Y - C	2.000 (-8.005, 12.005)	0.657
Assyrtiko	2022	Cluster weight	DI-Y - C	-0.000 (-73.619, 73.619)	1.000
Assyrtiko	2022	Cluster weight	NI-Y - C	1.202 (-72.417, 74.821)	0.971
Assyrtiko	2023	Cluster weight	DI-Y - C	-19.223 (-60.024, 21.578)	0.309
Assyrtiko	2023	Cluster weight	NI-Y - C	-27.390 (-68.191, 13.412)	0.160
Assyrtiko	2022	Cluster length	DI-Y - C	-1.261 (-4.092, 1.570)	0.334
Assyrtiko	2022	Cluster length	NI-Y - C	-0.344 (-3.175, 2.486)	0.786
Assyrtiko	2023	Cluster length	DI-Y - C	-1.778 (-4.912, 1.356)	0.227
Assyrtiko	2023	Cluster length	NI-Y - C	-0.361 (-3.495, 2.773)	0.797
Assyrtiko	2022	Cluster width	DI-Y - C	0.661 (-1.849, 3.172)	0.561
Assyrtiko	2022	Cluster width	NI-Y - C	-0.172 (-2.683, 2.338)	0.878
Assyrtiko	2023	Cluster width	DI-Y - C	-1.611 (-3.150, -0.072)	0.042
Assyrtiko	2023	Cluster width	NI-Y - C	-0.444 (-1.983, 1.094)	0.524
Assyrtiko	2022	Cluster compactness	DI-Y - C	0.598 (-3.080, 4.276)	0.718
Assyrtiko	2022	Cluster compactness	NI-Y - C	-0.195 (-3.872, 3.483)	0.906
Assyrtiko	2023	Cluster compactness	DI-Y - C	-4.590 (-6.094, -3.086)	<0.001
Assyrtiko	2023	Cluster compactness	NI-Y - C	-1.672 (-3.176, -0.167)	0.034
Assyrtiko	2022	Berry weight	DI-Y - C	0.316 (-0.108, 0.740)	0.124
Assyrtiko	2022	Berry weight	NI-Y - C	-0.809 (-1.233, -0.386)	0.002
Assyrtiko	2023	Berry weight	DI-Y - C	-0.109 (-0.313, 0.095)	0.254
Assyrtiko	2023	Berry weight	NI-Y - C	-0.452 (-0.656, -0.248)	<0.001
Assyrtiko	2022	Berry length	DI-Y - C	1.664 (0.421, 2.906)	0.015
Assyrtiko	2022	Berry length	NI-Y - C	-1.641 (-2.884, -0.398)	0.016

Cultivar	Year / Time	Variable	Contrast	Estimate (95% CI)	p
Assyrtiko	2023	Berry length	DI-Y - C	-0.168 (-0.936, 0.600)	0.628
Assyrtiko	2023	Berry length	NI-Y - C	-1.654 (-2.422, -0.886)	0.001
Assyrtiko	2022	Berry width	DI-Y - C	1.844 (0.856, 2.832)	0.003
Assyrtiko	2022	Berry width	NI-Y - C	-1.181 (-2.169, -0.193)	0.025
Assyrtiko	2023	Berry width	DI-Y - C	-0.894 (-1.604, -0.184)	0.020
Assyrtiko	2023	Berry width	NI-Y - C	-2.493 (-3.203, -1.784)	<0.001
Assyrtiko	2022	Relative skin mass	DI-Y - C	-3.018 (-5.081, -0.955)	0.010
Assyrtiko	2022	Relative skin mass	NI-Y - C	-5.394 (-7.458, -3.331)	<0.001
Assyrtiko	2023	Relative skin mass	DI-Y - C	-0.718 (-3.305, 1.869)	0.540
Assyrtiko	2023	Relative skin mass	NI-Y - C	-1.492 (-4.079, 1.095)	0.220
Assyrtiko	2022	Relative seed mass	DI-Y - C	-0.894 (-2.515, 0.726)	0.239
Assyrtiko	2022	Relative seed mass	NI-Y - C	0.020 (-1.601, 1.640)	0.978
Assyrtiko	2023	Relative seed mass	DI-Y - C	-0.461 (-0.859, -0.063)	0.028
Assyrtiko	2023	Relative seed mass	NI-Y - C	1.597 (1.199, 1.995)	<0.001

Berry composition

Cultivar	Year / Time	Variable	Contrast	Estimate (95% CI)	p
Agiorgitiko	2022	TSS	DI-Y - C	-1.037 (-1.773, -0.300)	0.012
Agiorgitiko	2022	TSS	NI-Y - C	-0.693 (-1.430, 0.043)	0.062
Agiorgitiko	2023	TSS	DI-Y - C	0.700 (-2.205, 3.605)	0.594
Agiorgitiko	2023	TSS	NI-Y - C	-1.767 (-4.671, 1.138)	0.198
Agiorgitiko	2022	TA	DI-Y - C	0.350 (0.174, 0.526)	0.002
Agiorgitiko	2022	TA	NI-Y - C	-0.163 (-0.338, 0.013)	0.065
Agiorgitiko	2023	TA	DI-Y - C	0.350 (-0.705, 1.405)	0.466
Agiorgitiko	2023	TA	NI-Y - C	0.550 (-0.505, 1.605)	0.264
Agiorgitiko	2022	pH	DI-Y - C	-0.092 (-0.145, -0.038)	0.004
Agiorgitiko	2022	pH	NI-Y - C	0.040 (-0.014, 0.094)	0.124
Agiorgitiko	2023	pH	DI-Y - C	0.147 (0.054, 0.240)	0.007

Cultivar	Year / Time	Variable	Contrast	Estimate (95% CI)	p
Agiorgitiko	2023	pH	NI-Y - C	0.047 (-0.046, 0.140)	0.280
Agiorgitiko	2022	TPC	DI-Y - C	0.604 (0.115, 1.094)	0.022
Agiorgitiko	2022	TPC	NI-Y - C	0.197 (-0.292, 0.687)	0.380
Agiorgitiko	2023	TPC	DI-Y - C	-0.049 (-0.466, 0.367)	0.792
Agiorgitiko	2023	TPC	NI-Y - C	0.096 (-0.320, 0.513)	0.609
Agiorgitiko	2022	Anthocyanins	DI-Y - C	0.219 (-0.175, 0.614)	0.236
Agiorgitiko	2022	Anthocyanins	NI-Y - C	0.214 (-0.180, 0.609)	0.246
Agiorgitiko	2023	Anthocyanins	DI-Y - C	0.169 (-0.068, 0.405)	0.139
Agiorgitiko	2023	Anthocyanins	NI-Y - C	0.267 (0.030, 0.504)	0.032
Assyrtiko	2022	TSS	DI-Y - C	-0.317 (-1.311, 0.678)	0.484
Assyrtiko	2022	TSS	NI-Y - C	-0.287 (-1.281, 0.708)	0.525
Assyrtiko	2023	TSS	DI-Y - C	-0.300 (-0.822, 0.222)	0.222
Assyrtiko	2023	TSS	NI-Y - C	-0.300 (-0.822, 0.222)	0.222
Assyrtiko	2022	TA	DI-Y - C	-0.338 (-0.629, -0.046)	0.028
Assyrtiko	2022	TA	NI-Y - C	0.612 (0.321, 0.904)	0.001
Assyrtiko	2023	TA	DI-Y - C	2.075 (1.401, 2.749)	<0.001
Assyrtiko	2023	TA	NI-Y - C	2.075 (1.401, 2.749)	<0.001
Assyrtiko	2022	pH	DI-Y - C	0.062 (-0.222, 0.346)	0.630
Assyrtiko	2022	pH	NI-Y - C	0.058 (-0.226, 0.342)	0.648
Assyrtiko	2023	pH	DI-Y - C	-0.337 (-0.370, -0.303)	<0.001
Assyrtiko	2023	pH	NI-Y - C	-0.337 (-0.370, -0.303)	<0.001
Assyrtiko	2022	TPC	DI-Y - C	0.362 (-0.051, 0.775)	0.078
Assyrtiko	2022	TPC	NI-Y - C	0.528 (0.114, 0.941)	0.019
Assyrtiko	2023	TPC	DI-Y - C	0.365 (-0.052, 0.782)	0.078
Assyrtiko	2023	TPC	NI-Y - C	0.530 (0.114, 0.947)	0.019

Gas exchange (marginal contrasts over measurement dates)

Cultivar	Year / Time	Variable	Contrast	Estimate (95% CI)	p
Agiorgitiko	Morning	A	DI-Y - C	-0.632 (-2.783, 1.519)	0.565
Agiorgitiko	Morning	A	NI-Y - C	-1.542 (-3.131, 0.047)	0.057
Agiorgitiko	Morning	E	DI-Y - C	-0.011 (-0.350, 0.329)	0.951
Agiorgitiko	Morning	E	NI-Y - C	-1.054 (-1.487, -0.621)	<0.001
Agiorgitiko	Morning	gs	DI-Y - C	-0.022 (-0.069, 0.025)	0.356
Agiorgitiko	Morning	gs	NI-Y - C	-0.084 (-0.131, -0.038)	<0.001
Agiorgitiko	Morning	WUEi	DI-Y - C	-1.389 (-11.542, 8.764)	0.789
Agiorgitiko	Morning	WUEi	NI-Y - C	14.125 (5.363, 22.887)	0.002
Agiorgitiko	Midday	A	DI-Y - C	-0.281 (-1.501, 0.938)	0.651
Agiorgitiko	Midday	A	NI-Y - C	-0.509 (-1.767, 0.749)	0.428
Agiorgitiko	Midday	E	DI-Y - C	0.321 (-0.837, 1.478)	0.587
Agiorgitiko	Midday	E	NI-Y - C	-0.002 (-1.212, 1.209)	0.998
Agiorgitiko	Midday	gs	DI-Y - C	-0.001 (-0.031, 0.029)	0.945
Agiorgitiko	Midday	gs	NI-Y - C	-0.005 (-0.035, 0.024)	0.720
Agiorgitiko	Midday	WUEi	DI-Y - C	-23.433 (-136.312, 89.445)	0.684
Agiorgitiko	Midday	WUEi	NI-Y - C	-7.122 (-117.704, 103.460)	0.900
Assyrtiko	Morning	A	DI-Y - C	-3.377 (-4.523, -2.230)	<0.001
Assyrtiko	Morning	A	NI-Y - C	-1.815 (-2.799, -0.831)	<0.001
Assyrtiko	Morning	E	DI-Y - C	-0.740 (-1.323, -0.157)	0.013
Assyrtiko	Morning	E	NI-Y - C	-0.345 (-1.014, 0.323)	0.311
Assyrtiko	Morning	gs	DI-Y - C	-0.109 (-0.172, -0.046)	<0.001
Assyrtiko	Morning	gs	NI-Y - C	-0.083 (-0.139, -0.027)	0.004
Assyrtiko	Morning	WUEi	DI-Y - C	22.645 (9.266, 36.023)	<0.001
Assyrtiko	Morning	WUEi	NI-Y - C	20.132 (8.349, 31.915)	<0.001
Assyrtiko	Midday	A	DI-Y - C	-1.328 (-4.131, 1.475)	0.353
Assyrtiko	Midday	A	NI-Y - C	-3.018 (-5.847, -0.189)	0.037
Assyrtiko	Midday	E	DI-Y - C	-0.567 (-2.133, 1.000)	0.478
Assyrtiko	Midday	E	NI-Y - C	-0.803 (-2.234, 0.627)	0.271

Cultivar	Year / Time	Variable	Contrast	Estimate (95% CI)	p
Assyrtiko	Midday	gs	DI-Y - C	-0.019 (-0.060, 0.022)	0.367
Assyrtiko	Midday	gs	NI-Y - C	-0.023 (-0.055, 0.009)	0.151
Assyrtiko	Midday	WUEi	DI-Y - C	15.496 (-97.007, 127.998)	0.787
Assyrtiko	Midday	WUEi	NI-Y - C	-53.565 (-75.770, -31.361)	<0.001

Leaf temperature (marginal contrasts over measurement dates)

Cultivar	Year / Time	Variable	Contrast	Estimate (95% CI)	p
Agiorgitiko	Morning	Tleaf	DI-Y - C	-1.828 (-4.167, 0.512)	0.126
Agiorgitiko	Morning	Tleaf	NI-Y - C	-2.900 (-4.949, -0.852)	0.006
Agiorgitiko	Midday	Tleaf	DI-Y - C	-1.392 (-2.939, 0.156)	0.078
Agiorgitiko	Midday	Tleaf	NI-Y - C	0.208 (-1.862, 2.279)	0.844
Assyrtiko	Morning	Tleaf	DI-Y - C	0.460 (-1.728, 2.648)	0.680
Assyrtiko	Morning	Tleaf	NI-Y - C	0.439 (-1.636, 2.513)	0.678
Assyrtiko	Midday	Tleaf	DI-Y - C	1.322 (-1.504, 4.148)	0.359
Assyrtiko	Midday	Tleaf	NI-Y - C	1.850 (-0.188, 3.888)	0.075
